# Development of a Bayesian multimodal model to detect biomarkers in neuroimaging studies

**DOI:** 10.3389/fnimg.2023.1147508

**Published:** 2023-05-24

**Authors:** Dulal K. Bhaumik, Yue Wang, Pei-Shan Yen, Olusola A. Ajilore

**Affiliations:** ^1^Division of Epidemiology and Biostatistics, University of Illinois at Chicago, Chicago, IL, United States; ^2^Department of Psychiatry, University of Illinois at Chicago, Chicago, IL, United States

**Keywords:** multiple testing, local false discovery rate, multimodal, functional connectivity, mixed-effects model

## Abstract

In this article, we developed a Bayesian multimodal model to detect biomarkers (or neuromarkers) using resting-state functional and structural data while comparing a late-life depression group with a healthy control group. Biomarker detection helps determine a target for treatment intervention to get the optimal therapeutic benefit for treatment-resistant patients. The borrowing strength of the structural connectivity has been quantified for functional activity while detecting the biomarker. In the biomarker searching process, thousands of hypotheses are generated and tested simultaneously using our novel method to control the false discovery rate for small samples. Several existing statistical approaches, frequently used in analyzing neuroimaging data have been investigated and compared via simulation with the proposed approach to show its excellent performance. Results are illustrated with a live data set generated in a late-life depression study. The role of detected biomarkers in terms of cognitive function has been explored.

## 1. Introduction

Late-life depression (LLD) is a common disorder associated with emotional distress, cognitive impairment, and somatic complaints in older people. Patients with LLD are usually over 55 years old and have major depressive symptoms. As the world population of adults aged 60 and older is expanding rapidly from 900 million in 2015 to 2 billion by 2050 (WHO, 2021), understanding age-related disorders is becoming prominent and necessary to accommodate this demographic shift. The general strategy of treating patients having major depressing disorder (MDD) or anxiety disorders with serotonin reuptake inhibitors has a success rate of 50–70% only, and there is no data to support therapeutic selection for these kinds of diseases (Dunlop et al., 2012). Therefore, the field of psychiatric research needs the discovery of biomarkers to guide treatment selection.

The objective of the article is to provide a guidance for treating LLD patients who require alternative treatments. This investigation aims to identify reliable disrupted brain regions (or biomarkers) in LLD patients that can be used to develop neuropsychotherapy, a safe and enriched environment utilizing a neurological approach to facilitate long-term change in neural function. Our approach detects reliable biomarkers, minimizing the risk of false detection, which is essential to get the optimal result from interventions. An immediate benefit of our approach is the development of interventions, such as cognitive behavioral therapy, that explore the association of the cognitive role of detected biomarkers with neurobehavioral measures. In addition, detected biomarkers can accurately predict prospective subjects at high risk for early prevention.

Brain connectivity usually refers to a pattern of anatomical links (structural connectivity) and statistical dependencies (functional connectivity). Structural connectivity (SC) is the structural link or neural pathways between two areas. This typically corresponds to white matter tracts that run between pairs of brain regions. Functional connectivity (FC) is defined as the “temporal correlation between spatially remote neurophysiological events (Friston et al., 1993).” In other words, FC indicates the synchronized and correlated patterns of activity. An essential problem in neural connectivity research is detecting disrupted connectivity for targeting treatment interventions to achieve optimal therapeutic benefit. The literature suggests that functional and structural abnormality are neurobiologically correlated in LLD (Tadayonnejad et al., 2014). It is a long-standing interest of researchers to know how FC and SC are related, whether FC is mediated by SC or another way. It is also equally important to investigate whether disruptions in neuro-connectivity can be detected with better accuracy by incorporating the information from functional and structural neuroimaging data. This article develops a Bayesian mixture model utilizing resting-state functional magnetic resonance imaging (rs-fMRI) and diffusion tensor imaging (DTI) data to detect disrupted brain region of interest (ROI) and use it as a biomarker while comparing two distinct groups (e.g., LLD and healthy control) of subjects.

Two significant contributions, namely, building a Bayesian mixture model for FC utilizing SC as auxiliary information and developing a strategy for multiple comparisons to control the false discovery rate (FDR), make this article novel for detecting biomarkers. Our Bayesian multimodal approach based on a Bayesian mixture model takes the advantage of the complementary SC data to enhance the modeling of the density of FC statistics while controlling the local false discovery rate (Lfdr; Efron, 2007). Our approach is general and helpful in a broad spectrum of applications with multiple modalities. The utility of our approach is illustrated with extensive simulations to show how it controls the FDR even with small samples under different correlation structures compared to some standard methods that solely consider FC. In a way, our approach attempts to answer whether SC is at all needed to detect biomarkers for functional activity, especially when the study sample size is small.

Biswal et al. (1995) first observed a high coherent synchronous correlation between the blood-oxygen-level dependence (BOLD) signals (i.e., FC) from the regions of the somatic motor system in the left and right hemispheres of the brain in healthy volunteers during resting conditions and demonstrated that BOLD signals reflect neural activities for both resting-state and task-state. Since then, rs-fMRI has been applied extensively in cognitive neuroscience research to study patients with neurological, mental, or psychiatric disorders. Studies show that using rs-fMRI can help identify disruptions in FC in various forms of neurological disorders such as Alzheimer's disease (Dai et al., 2015), psychiatric disorders including depression (Alexopoulos, 2019), and attention deficit hyperactivity disorder (Uddin et al., 2009). This is the rationale behind considering rs-fMRI as a valid measure of functional activity.

Despite all available significant technical advantages for the analysis of fMRI data, a few challenges remain open. First, we ask how to develop a statistical model for integrating multimodal neuroimaging data optimally. Previous neuroimaging studies involving FC and SC for quantitative/computational modeling (Honey et al., 2009) revealed that brain regions with substantial SC exhibit strong FC, but strong FC could also occur in regions with weak SC. These results suggest that strong SC can be a predictor for strong FC, but the opposite does not necessarily stands (i.e., weak SC cannot be a predictor for weak FC). Information provided by multimodal imaging techniques can complement each other, and thus integrated multimodal analysis enables us to borrow strength from different modalities. In a review paper of studies combining SC and FC data, Rykhlevskaia et al. (2008) discussed various approaches to integrate FC and SC, including analysis of FC informed by SC. Zhao (2014) employed a bivariate model that incorporates between-modality and within-subject correlations while jointly analyzing FC and SC. Xue et al. (2015) introduced a Bayesian multimodal approach for analyzing FC time series data incorporating SC into modeling. Chiang (2016) developed a Bayesian autoregressive model that combined multimodal neuroimaging data by integrating SC into the prior information for improving the inference on effective connectivity. Bhaumik et al. (2018b) proposed a bivariate linear mixed-effects model with random subject intercepts and heteroscedastic errors to analyze FC and SC data jointly. Zhang et al. (2019) introduced a covariate-adaptive method employing a mixture of the generalized linear model and Gaussian model to optimize the *p*-value threshold for multiple hypothesis testing and applied it to the fMRI data with Brodmann area (cerebral cortex regions) as the covariate. It remains challenging how to *optimally* utilize the information of SC when the interest is in FC for a two-group (e.g., disease and healthy control) comparison study.

Second, high-throughput (a faster method that can produce a greater number of samples to be processed in the same or less time) neuroimaging technologies generate an incredibly large amount of data, “big data,” which can be very challenging to analyze and interpret. The primary objective of neuroimaging studies for comparing two groups is to detect any differences in connectivity associated with the disease. As such, thousands of hypotheses are tested simultaneously, known as the large-scale simultaneous hypothesis testing problem (Efron, 2004). Most of these hypotheses are null, containing nothing but noise, while only a small number of hypotheses have true signals. This sparsity issue makes the problem of detecting true signals very challenging (Sun and Cai, 2007). Conventional statistical methods used for multiple testing to control the family-wise error rate tend to be overly conservative in large-scale hypothesis testing, leading to a high chance of missing many important significant hypotheses that may be meaningful to researchers. An effective way of controlling FDR is needed for bimodal neuroimaging studies.

Third, the current economic cost of imaging techniques is high; consequently, most neuroimaging studies have a relatively limited number of subjects. Szucs and Ioannidis (2019) conducted a systematic review and evaluated sample sizes across highly cited published MRI study papers between 1990 and 2012 and reported a median sample size of 12.5 per group based on 107 clinical fMRI studies with more than one group. The high sampling variability associated with a small sample leads to lower power with an inflated probability of falsely detected significant findings. Existing statistical methodologies hardly provide satisfactory results in controlling the FDR for neuroimaging studies with small samples (Bhaumik et al., 2018a). Therefore, developing a novel statistical method with a small sample to control the FDR better is critically important and challenging.

We address all three aforementioned concerns while detecting biomarkers. This article is structured as follows. In Section 2, we introduce a neuroimaging study in LLD, the motivating example for this research, where FC and SC data were collected from each participant. In Section 3, we develop a Bayesian multimodal Lfdr method (BLfdr) utilizing a Bayesian mixture model that integrates FC and SC data. This is followed by extensive simulations designed to evaluate the performance of our proposed method in Section 4. Further, we compare our simulated results with Efron's Lfdr method, which solely considers FC. In Section 5, we compare our results using BLfdr with Lfdr and Dirichlet process mixture model (DPM) in the context of the motivating example. Detailed results involving major brain networks associated with LLD are discussed. While classifying subjects to the LLD group, the excellent performance of BLfdr compared to other methods is shown in a table. In Section 6, we compare our approach with another multimodal method to detect biomarkers. In Section 7, we discuss how our discovery can be used to treat LLD subjects and some directions for future work.

## 2. Motivational example

In order to understand the relationship between structural abnormality and altered functional brain networks in LLD, the principal investigator Ajilore and his team conducted a study at the University of Illinois at Chicago (UIC). The study was approved by UIC Institutional Review Board and performed in compliance with the Declaration of Helsinki. Ajilore's study selected ten unmedicated and symptomatic subjects with geriatric depression (referred to as the LLD group) and 13 healthy elderly comparison subjects (referred to as the HC group) from the community. A total of 87 ROIs were parcelated by the Freesurfer Desikan atlas (Desikan et al., 2006). The study aimed to examine the relationship between abnormal structural changes in patients with LLD and whole-brain FC alternations. Study results are reported in a research paper (Rykhlevskaia et al., 2008). This study motivates us to develop an appropriate statistical methodology needed to achieve the goal.

## 3. Methods

Our Bayesian multimodal model utilizes the complementary SC data to enhance the modeling of the density of FC statistics while controlling the Lfdr. We assessed a late-fusion approach (Ramachandram and Taylor, 2017) by collecting individual resting-state average correlations of pairwise BOLD signal for FC and fiber counts for SC. The FC was measured using the FC toolbox CONN (Whitfield-Gabrieli and Nieto-Castanon, 2012), which performs seed-based correlation analysis by computing the Pearson correlation coefficients between the BOLD time series from a given ROI to all other ROIs in the brain. Prior to data analysis, potential confounding factors including motion artifact, white matter, cerebrospinal fluid, and physiological noise source reduction were regressed out from the signal. Pearson correlation was then transformed into an approximately normal distribution using Fisher's *Z* transformation (Bartlett, 1993). A link-specific test statistic was developed to compare the corresponding links of the two groups. Three specific examples of such test statistics are (i) t-statistic to compare two correlations (from two groups) for a link (Rykhlevskaia et al., 2008), (ii) z-statistic to compare two Fisher-*Z* transformed correlations, and (iii) t-statistic to compare two intercepts of the linear mixed-effects model (Bhaumik et al., 2018a). In what follows, we first briefly describe Efron's Lfdr approach and then develop our Bayesian Mixture Model.

### 3.1. Bayesian multimodal local false discovery rate

FDR has been used to address the multiple testing issue in neuroimaging studies where it is defined as the expected proportion of false positives among all rejected hypotheses (Benjamini and Hochberg, 2000). The concept of FDR was further extended from an empirical Bayesian perspective (Efron, 2007). The Lfdr is defined as the posterior probability of the true null hypothesis given the observed test statistics. Literature suggests that Efron's Lfdr method performs better than other popular approaches for multiple comparisons in neuroimaging studies (Bhaumik et al., 2018a,b). What makes this problem exciting and challenging is whether extra strength can be borrowed from SC to better control the FDR by extending the Lfdr approach while analyzing FC data. In this article, we plan to develop a Bayesian multimodal approach to control the FDR extending the basic concept of Lfdr.

In Efron's Lfdr procedure, the status of the ith hypothesis is denoted by *H*_0,*i*_. Assume a total of *m* hypotheses are tested simultaneously (i.e., *i* = 1, 2, ..., *m*). Let *H*_0,*i*_ = 0 for a true *H*_0,*i*_, and *H*_0,*i*_ = 1 otherwise, and *t*_*i*_ be the corresponding test statistic. *H*_0,*i*_ follows i.i.d. Bernoulli distribution with probability *p*_1_. The Bayesian two-group mixture model (Efron, 2008) is given by,

(1) $$\begin{array}{l} f\left(t_{i}\right)=p_{0}f_{0}\left(t_{i}\right)+p_{1}f_{1}\left(t_{i}\right), \end{array}$$

where *p*_0_ = *P*(*H*_0,*i*_ = 0) and *p*_1_ = *P*(*H*_0,*i*_ = 1) = 1 − *p*_0_ are the prior probabilities that are assumed to be the same across all hypotheses, *f*_0_ and *f*_1_ are the probability density functions of the test statistic *t*_*i*_ under null and alternative hypotheses, respectively. The Lfdr with *t*_*i*_ is defined as

(2) $$\begin{array}{l} Lfdr_{i}\left(t_{i}\right)=P\left(H_{0,i}=0|T=t_{i}\right)=\frac{p_{0}f_{0}\left(t_{i}\right)}{f\left(t_{i}\right)}, \end{array}$$

where *p*_0_, *f*_0_(*t*), and *f*(*t*) can be estimated using the Poisson generalized linear model and “central matching” method based on the “zero assumption” that the central peak of the distribution of test statistics is comprised mostly of the null hypotheses (Efron, 2004, 2007). We extend the covariate-modulated Lfdr method integrating SC and FC statistics to improve the FDR for a cross-sectional, multimodal neuroimaging study with a small sample size (Zablocki et al., 2014). Our Bayesian multimodal Lfdr approach has two distinct features. First, for each FC link, the prior probability is modeled using a logistic regression model with corresponding link-specific SC statistics as a covariate instead of assuming a constant *p*_0_ for all FC links in the Lfdr method that solely considers FC. Second, the densities of FC test statistics under null and alternative hypotheses are estimated using parametric models, where the posterior sampling for the model parameters is obtained using Gibbs sampling. The SC information serves as auxiliary information in the mixture model, aiding the identification of difference of FC between the disease and control group.

### 3.2. Bayesian mixture model

Under “zero assumption,” most of the test statistics around zero are from the null hypothesis. There may not be a sufficient number of test statistics under alternative hypotheses in either lower or upper tail areas that can be used for parametric density estimation. Thus, combining both tail areas provides better information to estimate the alternative density. Therefore, we use absolute values of test statistics compromising directions for modeling the density under alternative hypotheses.

From both theoretical and empirical perspectives, it is assumed that the underlying distribution of test statistics under the null hypothesis is normal, as described in the previous section (Efron, 2004, 2007). Under the null hypothesis, absolute values of test statistics follow a folded normal distribution, in which the probability mass values on the left half of the normal distribution are folded over the right half, as “folded” literally means.

Let $t_{i}^{\left(F\right)}$ and $t_{i}^{\left(S\right)}$ denote the absolute values of test statistics for FC and SC, respectively, to compare mean connectivity measures between the disease group and control group for the *i*^*th*^ connectivity link (*i* = 1, ⋯ , *m*). The $t_{i}^{\left(F\right)}$ and $t_{i}^{\left(S\right)}$ statistics are obtained by fitting the FC and SC data using a univariate linear mixed-effects regression model with heteroscedastic errors (Bhaumik et al., 2018a,b), separately. And $t^{\left(F\right)}={\left[\begin{array}{cccc} t_{1}^{\left(F\right)} & t_{2}^{\left(F\right)} & \cdots & t_{m}^{\left(F\right)} \end{array}\right]}^{T}$and $t^{\left(S\right)}={\left[\begin{array}{cccc} t_{1}^{\left(S\right)} & t_{2}^{\left(S\right)} & \cdots & t_{m}^{\left(S\right)} \end{array}\right]}^{T}$are the vectors of the absolute values of test statistics for FC and SC, respectively.

Assume that for links under the null hypothesis of no FC difference between two groups (referred to as null links), the corresponding test statistic $t_{i}^{\left(F\right)}$ follows a folded normal distribution (*f*_0_) with zero location parameter (μ_0_ = 0) and unknown scale parameter ($\sigma_{0}^{2}>0$). The density function *f*_0_ of the null FC has the following expression.

(3) $$\begin{array}{lll} f_{0}\left(t_{i}^{\left(F\right)}|\mu_{0}=0,\sigma_{0}^{2}\right) & = & \frac{1}{\sqrt{2\pi \sigma_{0}^{2}}}\exp\left[-\frac{{\left(t_{i}^{\left(F\right)}-\mu_{0}\right)}^{2}}{2\sigma_{0}^{2}}\right]\\ \;+\frac{1}{\sqrt{2\pi \sigma_{0}^{2}}}\exp\left[-\frac{{\left(t_{i}^{\left(F\right)}+\mu_{0}\right)}^{2}}{2\sigma_{0}^{2}}\right], \end{array}$$

For a comparison study with two groups, the zero mean assumption under the null hypothesis is justified, and the $\sigma_{0}^{2}$ assumption provides better flexibility to explain variability. Note that the SC test statistic $t_{i}^{\left(S\right)}$ is not involved in (3), under the assumption that SC statistic has an insignificant influence on the null density of FC statistics (Zhang et al., 2019).

Next, we assume that for links with significant FC differences between two groups (referred to as alternative links), the corresponding test statistic $t_{i}^{\left(F\right)}$ follows a gamma distribution (*f*_1_) with a fixed location parameter μ_1_, unknown shape parameter $\alpha \left(t_{i}^{\left(S\right)}|\alpha \right)>0$ and rate parameter β > 0. We consider a gamma distribution because of its flexibility in accommodating the skewness and flatness in the density function. Denote the shape parameter of the gamma distribution by α, given the *t*-value $t_{i}^{\left(S\right)}$ of SC and parameter vector ***α***, where $\alpha =\alpha \left(t_{i}^{\left(S\right)}|\alpha \right)=\exp\left(\alpha_{0}+\alpha_{1}t_{i}^{\left(S\right)}\right)$. The density function of *f*_1_ is as follows.

(4) $$\begin{array}{ll} f_{1}\left(t_{i}^{\left(F\right)}|\mu_{1},\alpha ),\beta \right) & =\frac{\beta^{\alpha \left(t_{i}^{\left(S\right)}|\alpha \right)}}{\Gamma \left(\alpha \left(t_{i}^{\left(S\right)}|\alpha \right)\right)}{\left(t_{i}^{\left(F\right)}-\mu_{1}\right)}^{\alpha \left(t_{i}^{\left(F\right)}|\alpha \right)-1}\\ \;\exp\left[-\beta \left(t_{i}^{\left(F\right)}-\mu_{1}\right)\right]. \end{array}$$

Further, we assume that $\alpha \left(t_{i}^{\left(S\right)}|\alpha \right)$ is a log-linear function of SC statistic $t_{i}^{\left(S\right)}$ with unknown parameters $\alpha ={\left[\begin{array}{cc} \alpha_{0} & \alpha_{1} \end{array}\right]}^{T}$ and $\alpha \left(t_{i}^{\left(S\right)}|\alpha \right)=\exp\left(\alpha_{0}+\alpha_{1}t_{i}^{\left(S\right)}\right)$. To avoid the non-convergence issue and gain some efficiency, we assume β does not depend on $t_{i}^{\left(S\right)}$. The location parameter μ_1_ in (4) is purposely fixed at 0.674, which is the median of the standard folded normal distribution. Based on the “zero assumption,” it is reasonable to assume that $t_{i}^{\left(F\right)}\leq 0.674$ is null *a priori*, so the alternative density is bounded away from zero. Also, this restriction on μ_1_ in *f*_1_ is necessary to address the identifiability issue when strong assumptions on parameters are unavailable.

To complete the mixture model, we define a latent variable *w*_*i*_ to indicate which component of the mixture model (*f*_0_ for null and *f*_1_ for alternative) is used as a density function for each $t_{i}^{\left(F\right)}$. The prior probability of *w*_*i*_ = 1 denoted by $\pi_{1}\left(t_{i}^{\left(S\right)}\right)$ and modeled using a logistic regression with $t_{i}^{\left(S\right)}$ as a covariate,

(5) $$\begin{array}{l} \pi_{1}\left(t_{i}^{\left(S\right)}|\gamma \right)=P\left(w_{i}=1|t_{i}^{\left(S\right)}\right)=\frac{\exp\left(\gamma_{0}+\gamma_{1}t_{i}^{\left(S\right)}\right)}{1+\exp\left(\gamma_{0}+\gamma_{1}t_{i}^{\left(S\right)}\right)}, \end{array}$$

where $\gamma ={\left[\begin{array}{cc} \gamma_{0} & \gamma_{1} \end{array}\right]}^{T}$ is a vector of unknown parameters. The prior probability of *w*_*i*_ = 0, denoted by $\pi_{0}\left(t_{i}^{\left(S\right)}\right)$, is

(6) $$\begin{array}{lll} \pi_{0}\left(t_{i}^{\left(S\right)}|\gamma \right) & = & P\left(w_{i}=0|t_{i}^{\left(S\right)}\right)=1-\pi_{1}\left(t_{i}^{\left(S\right)}|\gamma \right)\\ \;=\frac{1}{1+\exp\left(\gamma_{0}+\gamma_{1}t_{i}^{\left(S\right)}\right)}. \end{array}$$

Then, the density of the mixture model (*f*) and the likelihood function $L=L\left(\alpha ,\gamma ,\beta ,\sigma_{0}^{2}|w,t^{\left(F\right)},t^{\left(S\right)}\right)$ are defined as follows, respectively,

(7) $$\begin{array}{l} f\left(t_{i}^{\left(F\right)}|t_{i}^{\left(S\right)},\alpha ,\gamma ,\beta ,\sigma_{0}^{2}\right)=\pi_{0}\left(t_{i}^{\left(S\right)}|\gamma \right)f_{0}\left(t_{i}^{\left(F\right)}|\sigma_{0}^{2}\right)\\ \;+\pi_{1}\left(t_{i}^{\left(S\right)}|\gamma \right)f_{1}\left(t_{i}^{\left(F\right)}|\alpha ,\beta ,t_{i}^{\left(S\right)}\right), \end{array}$$

(8) $$\begin{array}{l} L=\overset{m}{\underset{i=1}{\prod }}\{{\left[\pi_{0}\left(t_{i}^{\left(S\right)}|\gamma \right)f_{0}\left(t_{i}^{\left(F\right)}|\sigma_{0}^{2}\right)\right]}^{\left(1-w_{i}\right)}\\ {\left[\pi_{1}\left(t_{i}^{\left(S\right)}|\gamma \right)f_{1}\left(t_{i}^{\left(F\right)}|\alpha ,\beta ,t_{i}^{\left(S\right)}\right)\right]}^{w_{i}}\}, \end{array}$$

where $w={\left[\begin{array}{cccc} w_{1} & w_{2} & \cdots & w_{m} \end{array}\right]}^{T}$ is a vector of *w*_*i*_'s. The posterior probability (denoted by $\hat{BLfdr_{i}}\left(t_{i}^{\left(F\right)},t_{i}^{\left(S\right)}\right)$) of the ith null connectivity link given $t_{i}^{\left(F\right)}$, $t_{i}^{\left(S\right)}$ statistics is computed using Bayes' theorem with posterior estimates of the parameters $\left\{\hat{\alpha },\hat{\gamma },\hat{\beta },{\hat{\sigma }}_{0}^{2}\right\}$.

(9) $$\begin{array}{lll} \hat{BLfdr_{i}} & = & \left\{\pi_{0}\left(t_{i}^{\left(S\right)}|\hat{\gamma }\right)f_{0}\left(t_{i}^{\left(F\right)}|{\hat{\sigma }}_{0}^{2}\right)\right\}\{\pi_{0}\left(t_{i}^{\left(S\right)}|\hat{\gamma }\right)f_{0}\left(t_{i}^{\left(F\right)}|{\hat{\sigma_{0}}}^{2}\right)\\ \;+\pi_{1}\left(t_{i}^{\left(S\right)}|\hat{\gamma }\right)f_{1}\left(t_{i}^{\left(F\right)}|\hat{\alpha },\hat{\beta },t_{i}^{\left(S\right)}\right){\}}^{-1}. \end{array}$$

Comparing the right hand expression of (9) with that of (2), we see that the numerator of (9) has a weight function $\pi_{0}\left(t_{i}^{\left(S\right)}|\hat{\gamma }\right)$ that depends on SC. The denominator is a convex function of $f_{0}\left(t_{i}^{\left(F\right)}|{\hat{\sigma }}_{0}^{2}\right)$ and $f_{1}\left(t_{i}^{\left(F\right)}|\hat{\alpha },\hat{\beta },t_{i}^{\left(S\right)}\right)$, where *f*_1_ is a function of SC. Thus, $\hat{BLfdr_{i}}\left(t_{i}^{\left(F\right)},t_{i}^{\left(S\right)}\right)$ in (9) incorporates the information from SC at each link level, whereas *Lfdr*_*i*_(*t*_*i*_) in (2) does not depend on SC and related weights *p*_0_ and *p*_1_ are fixed for all links.

#### 3.2.1. Prior distributions

Gamma (0.001, 0.001) and Inverse-Gamma (0.001, 0.001) are commonly used as non-informative priors in Bayesian inference using Gibbs Sampling (Lunn et al., 2009). However, some concerns are being raised and discussed regarding these priors. First, these priors are improper as they do not have corresponding distribution functions. Second, those priors are unbounded, and the integral over the entire parameter space is infinite.

(10) $$\begin{array}{l} \int_{0}^{\infty }f\left(\theta \right)d\theta =\int_{0}^{\infty }\frac{1}{\theta }d\theta =\text{ln}\theta {|}_{0}^{\infty }=\infty -\left(-\infty \right)=\infty . \end{array}$$

**Figure 1:**
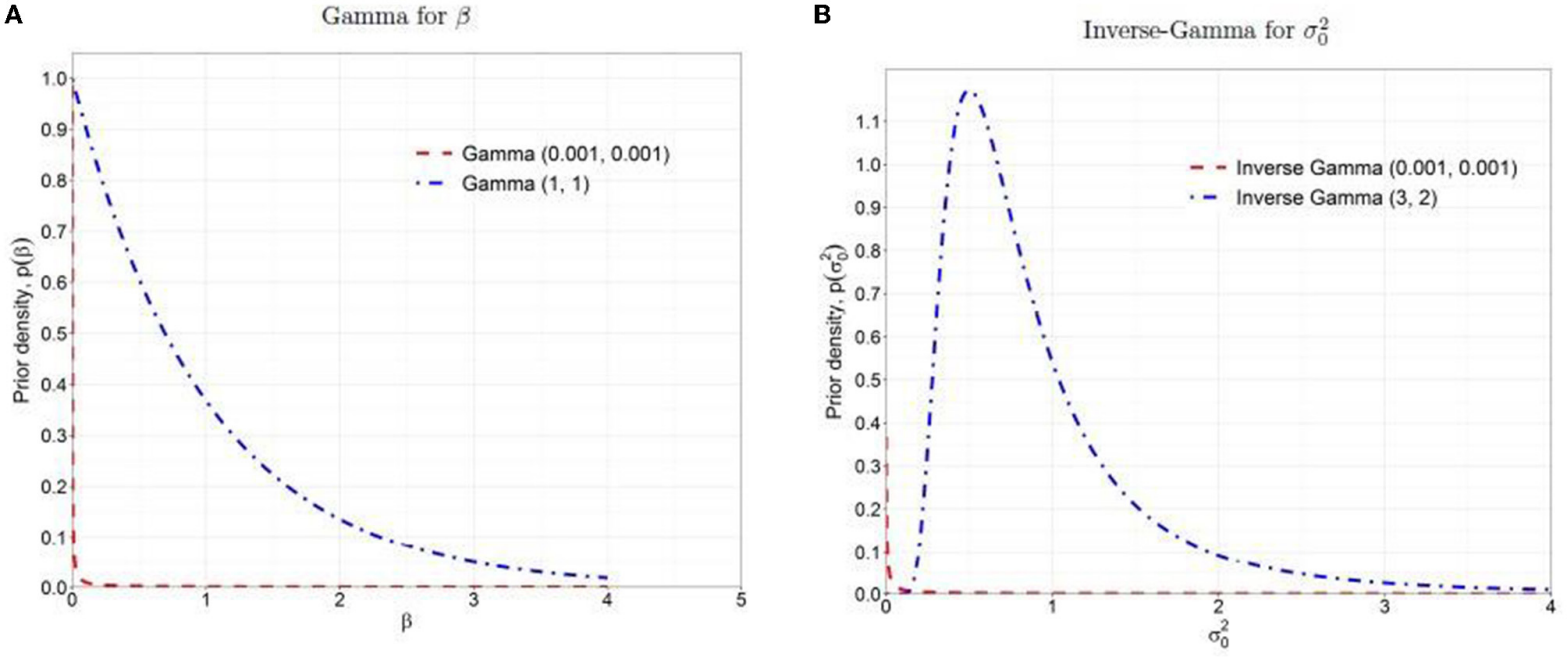
Prior distributions: **(A)** Gamma for β, **(B)** Inverse-Gamma for $\sigma_{0}^{2}$. The dashed red lines are the non-informative priors, and the dash-dotted blue lines are the weakly informative priors.

Improper priors also yield improper posterior densities (Gelman, 2006; Gelman et al., 2013). The gamma and inverse gamma prior distributions mentioned earlier have means of one and large variances with a high probability density close to zero and a very long tail (as shown in the dashed red lines in Figure 1). The high prior probability density near zero strongly influences the posterior density, leading to a posterior distribution similar to the prior, with a majority mass near zero and a long and heavy tail. As a result, the posterior distribution is unregularized and diffuses to extremely large values. In that sense, the priors are not non-informative but quite influential. Furthermore, in complex cases, the improper prior often results in non-convergence issues in the Gibbs sampler that draws samples directly from the conditional distributions (Robert et al., 1999).

Due to the undesirable properties of non-informative priors as discussed above, it is recommended to use weakly informative prior that contains a little but sufficient information to provide regularization and ensures that the posterior remains bounded within a reasonable range (Gelman, 2006; Gelman et al., 2008, 2013). In this study, we followed the concept of the method of moments estimation (Kim et al., 2009) and chose hyperparameters, Gamma (*a*_β_ = 1, *b*_β_ = 1) and Inverse-Gamma (*a*_σ_0__ = 3, *b*_σ_0__ = 2) with means = 1 and variances = 1 as the weakly informative priors for β and $\sigma_{0}^{2}$, respectively (as shown in the dash-dotted blue lines in Figure 1). These weakly informative priors offer more stable inferences than non-informative priors by providing regularization in parameter estimation and enough vagueness to ensure that the data dominate posteriors.

For the other unknown parameters {***α***, ***γ***} in the mixture model (7), we assume generic weakly informative prior distributions providing more stable results compared to non-informative prior, but still have enough vagueness to ensure that the data dominate the posteriors. For $\alpha ={\left[\begin{array}{cc} \alpha_{0} & \alpha_{1} \end{array}\right]}^{T}$and $\gamma ={\left[\begin{array}{cc} \gamma_{0} & \gamma_{1} \end{array}\right]}^{T}$, we assume that α_0_ and α_1_ are *a priori* independent, and also γ_0_ and γ_1_ are *a priori* independent, and their values are concentrated between −1 and 1. Further we assume ***α*** ~ *N*_2_(**0**, Σ_***α***_) and ***γ*** ~ *N*_2_(**0**, Σ_***γ***_), where $\Sigma_{\alpha }=\Sigma_{\gamma }=\left[\begin{array}{cc} 1 & 0\\ 0 & 1 \end{array}\right]$.

#### 3.2.2. Posterior distributions

We obtain the posterior sampling distributions for unknown parameters from Markov Chain Monte Carlo (MCMC) using Gibbs sampler and Metropolis-Hasting algorithm (Robert et al., 1999). Expressions of posterior distributions are:

(11) $$\begin{array}{l} f\left(\alpha |\beta ,w,t^{\left(F\right)},t^{\left(S\right)}\right)\propto \exp\left\{-\frac{1}{2}\alpha^{T}\Sigma_{\alpha }^{-1}\alpha \right\}\\ \;\underset{\left\{i|w_{i}=1\right\}}{\prod }\frac{\beta^{\alpha \left(t_{i}^{\left(S\right)}|\alpha \right)}}{\Gamma (\alpha \left(t_{i}^{\left(S\right)}|\alpha \right))}{\left(t_{i}^{\left(F\right)}-\mu_{1}\right)}^{\alpha \left(t_{i}^{\left(S\right)}|\alpha \right)}, \end{array}$$

(12) $$\begin{array}{l} f\left(\gamma |w,t^{\left(F\right)}\right)\propto \exp\left\{-\frac{1}{2}\gamma^{T}\Sigma_{\gamma }^{-1}\gamma \right\}\overset{m}{\underset{i=1}{\prod }}\frac{{\exp\left(\gamma_{0}+\gamma_{1}t_{i}^{\left(S\right)}\right)}^{w_{i}}}{1+\exp\left(\gamma_{0}+\gamma_{1}t_{i}^{\left(S\right)}\right)}, \end{array}$$

(13) $$\begin{array}{l} f\left(\beta |\alpha ,w,t^{\left(F\right)},t^{\left(S\right)}\right)\sim \text{Gamma}(1+\underset{\left\{i|w_{i}=1\right\}}{\sum }\alpha \left(t_{i}^{\left(S\right)}|\alpha \right),\;1\\ \;+\underset{\left\{i|w_{i}=1\right\}}{\sum }\left(t_{i}^{\left(F\right)}-\mu_{1}\right)), \end{array}$$

(14) $$\begin{array}{l} f\left(\sigma_{0}^{2}|w,t^{\left(F\right)}\right)\sim \text{Inverse-Gamma}(3+\frac{1}{2}\overset{m}{\underset{i=1}{\sum }}I_{\left\{w_{i}=0\right\}},2\\ \;+\frac{1}{2}\underset{\left\{i|w_{i}=0\right\}}{\sum }\left(t_{i}^{\left(F\right)}{)}^{2}\right). \end{array}$$

where $\alpha \left(t_{i}^{\left(S\right)}|\alpha \right)=\exp\left(\alpha_{0}+\alpha_{1}t_{i}^{\left(S\right)}\right)$, μ_1_ = 0.674, *I*_{_*w*__*i*_ = 0}_ = 1 if *w*_*i*_ = 0 and 0 otherwise.

The posterior sampling for β and $\sigma_{0}^{2}$ are directly drawn from the full conditional distributions (13) and (14), respectively. For ***α*** and ***γ***, posterior samplings are drawn from a multivariate *t* distribution with a small number of df ν such as ν = 4, providing three finite moments to approximate the density. Specifically, a multiple-try Metropolis algorithm is employed to increase the step size and acceptance rate (Liu et al., 2000).

For Markov chains, the initial values are set as $\alpha^{\left(0\right)}={\left[\begin{array}{cc} 0 & 0 \end{array}\right]}^{T}$, β^(0)^ = 0.1, $\sigma_{0}^{2(0)}=1$ and $\pi_{0}^{\left(0\right)}=0.94$. Denote the threshold that corresponds to $\pi_{0}^{\left(0\right)}=0.94$ by λ^(0)^, $w_{i}^{\left(0\right)}=1$ if $t_{i}^{\left(F\right)}\geq \lambda^{\left(0\right)}$, and 0 otherwise. Then, we can get the estimates of the coefficients based on the logistic regression model on $w_{i}^{\left(0\right)}$ given $t_{i}^{\left(S\right)}$, as the initial values of $\gamma^{\left(0\right)}={\left[\begin{array}{cc} \gamma_{0}^{\left(0\right)} & \gamma_{1}^{\left(0\right)} \end{array}\right]}^{T}$. At the *k*th MCMC iteration, *k* = 1, ..., *K*, when $\left\{\alpha^{\left(k\right)},\gamma^{\left(k\right)},\beta^{\left(k\right)},{\sigma_{0}^{2}}^{\left(k\right)}\right\}$ are drawn, the probability of the latent variable $w_{i}^{\left(k\right)}=1$ given $t_{i}^{\left(F\right)}$, $t_{i}^{\left(S\right)}$ and $\left\{\alpha^{\left(k\right)},\gamma^{\left(k\right)},\beta^{\left(k\right)},{\sigma_{0}^{2}}^{\left(k\right)}\right\}$ denoted by $p_{i}^{\left(k\right)}$, is updated using

(15) $$\begin{array}{ll} p_{i}^{\left(k\right)} & =P\left(w_{i}=1|t_{i}^{\left(F\right)},t_{i}^{\left(S\right)},\alpha^{\left(k\right)},\gamma^{\left(k\right)},\beta^{\left(k\right)},\sigma_{0}^{2(k)}\right)\\ \;=\left\{\pi_{1}\left(t_{i}^{\left(S\right)}|\gamma^{\left(k\right)}\right)f_{1}\left(t_{i}^{\left(F\right)}|t_{i}^{\left(S\right)},\alpha^{\left(k\right)},\beta^{\left(k\right)}\right)\right\}\\ \;\times \{\pi_{0}\left(t_{i}^{\left(S\right)}|\gamma^{\left(k\right)}\right)f_{0}\left(t_{i}^{\left(F\right)}|{\sigma_{0}^{2}}^{\left(k\right)}\right)\\ \;+\pi_{1}\left(t_{i}^{\left(S\right)}|\gamma^{\left(k\right)}\right)f_{1}\left(t_{i}^{\left(F\right)}|t_{i}^{\left(S\right)},\alpha^{\left(k\right)},\beta^{\left(k\right)}\right){\}}^{-1}. \end{array}$$

$w_{i}^{\left(k\right)}$ is sampled from the full conditional Bernoulli distribution with probability $p_{i}^{\left(k\right)}$, i.e., $w_{i}^{\left(k\right)}\overset{iid}{\sim }\;Bernoulli\;\left(p_{i}^{\left(k\right)}\right)$.

After obtaining samples for each parameter from their corresponding posterior distributions, we compute the posterior medians as the posterior estimates of the parameters $\left\{\hat{\alpha },\hat{\gamma },\hat{\beta },{\hat{\sigma }}_{0}^{2}\right\}$. The posterior probability that the *i*th connectivity link is null, given $t_{i}^{\left(F\right)}$ and $t_{i}^{\left(S\right)}$, denoted by ${\hat{BLfdr}}_{i}\left(t_{i}^{\left(F\right)},t_{i}^{\left(S\right)}\right)$. To ensure that the average FDR is controlled at a pre-specified level of *q*, we then apply the oracle procedure (Sun and Cai, 2007), which regards multiple testing as a compound decision problem and intended to minimize the false non-discovery rate that is subject to a constraint on the FDR ≤ *q*: (1) Sort the calculated ${\hat{BLfdr}}_{i}$, *i* = 1, ⋯ , *m* in ascending order denoted by ${\hat{BLfdr}}_{\left(1\right)}\leq {\hat{BLfdr}}_{\left(2\right)}\leq \cdots {\hat{BLfdr}}_{\left(m\right)}$; (2) For a given *q* (0 < *q* < 1), find *k* for which $k=\underset{i}{\arg\max}\left\{i:\frac{1}{i}\overset{i}{\underset{j=1}{\sum }}{\hat{BLfdr}}_{\left(j\right)}\leq q\right\}$; (3) Reject all *H*_(*i*)_, *i* = 1, ⋯ , *k*.

## 4. Simulation study

We performed a simulation study to see how the proposed Bayesian multimodal approach controls the local false discovery rate. Related algorithms, the combination of parametric space, and corresponding results are given below.

### 4.1. An algorithm for simulation

Several data sets are generated with varying sample sizes *n* = 15, 25, 35, and 45, following a very similar structure to that of the motivating LLD study. The performance of BLfdr is compared with that of the Lfdr method. To estimate the percentage of null (i.e., no significant difference between two groups) of FC, we computed a t-statistic utilizing the difference of Fisher's *Z* transformed Pearson correlations of LLD and HC. The corresponding *p*-value for each link is determined from the *t*-test statistic. Smaller *p*-values (< 0.05) are used to screen the first level of the null FC. Similarly, a Poisson distribution is used to detect a null link for SC.

We assume out of a total of 3,741 links, 1% links are non-null in both FC and SC, 1% links are non-null in FC, but those are null in SC, another 1% links are null in FC, but those are non-null in SC, and the rest 97% are null in both FC and SC. The 2% proportion of alternative links in FC aligns with the assumption used in the simulation study with the same LLD neuroimaging data (Song, 2016; Bhaumik et al., 2018b). The between-group difference is measured by the difference between the intercept parameters of a mixed-effects model while comparing LLD with HC. We consider the between-group difference of FC and SC for the alternative links as δ^(*F*)^ = 0.075 and δ^(*S*)^ = 0.25, respectively. To account for the potential correlation between FC and SC, we use a bivariate mixed-effects model with heteroscedastic errors to simulate FC and SC data jointly, where the parameters of the model are set close to the estimated values of those determined from our LLD data discussed before. A detailed description of the simulation strategy with various steps of selection of parameters is given in the Appendix.

Note that our assumption on errors is wide open by employing a gamma distribution to accommodate heterogeneity in links and skewness of the distribution. We make a realistic assumption that alternatives may vary following a gamma distribution, unlike a fixed alternative. Further, we assume that error distributions of FC and SC of HC are different from respective distributions of LLD. As SC and FC are nested within the same subject, it is natural to expect that those are correlated, which is reflected in our assumption.

### 4.2. Simulated results

**Figure 2:**
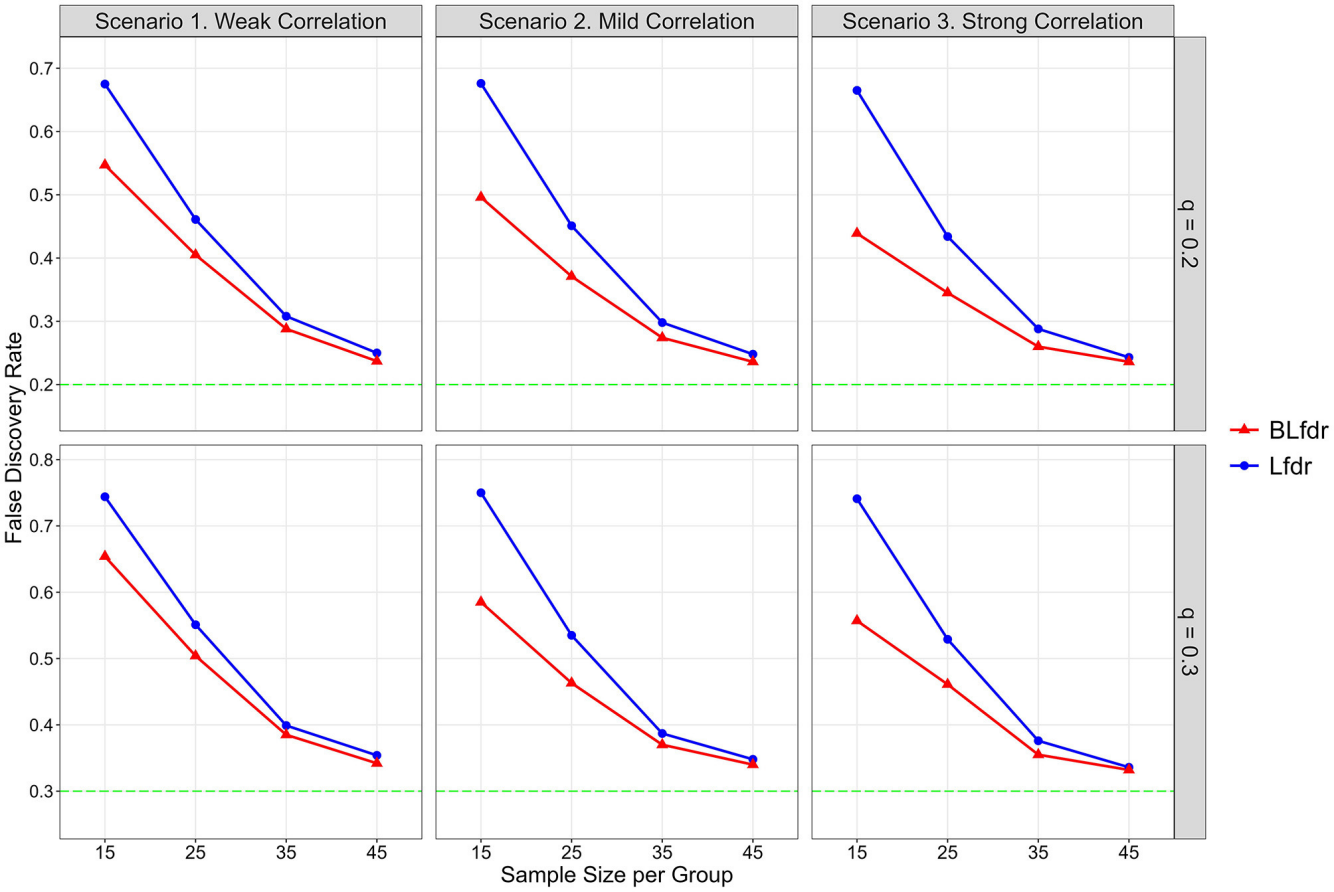
Simulated FDR by Lfdr and BLfdr. Results are based on 10,000 simulated data sets for each scenario assuming the mean FC difference between the LLD group and HC group for alternative links is δ^(*F*)^ = 0.075 and mean SC difference is δ^(*S*)^ = 0.25. For each simulation, δ^(*F*)^ is drawn from a uniform distribution *U*(0.055, 0.095) with a mean of 0.075, and δ^(*S*)^ is drawn from another uniform distribution *U*(0.15, 0.35) with a mean of 0.25. Each simulated data is fitted with a mixed-effects model to obtain test statistics for the 3,741 links for FC and SC separately. Simulated results are obtained from 80% data after deleting the top 10% and bottom 10% test statistics.

We present simulated results in Figure 2 in terms of FDR at pre-specified levels of *q* = 0.2 and 0.3 for a weak correlation = 0.1, mild correlation = 0.4, and strong correlation = 0.9. As mentioned before, neuroimaging studies usually have a small number of subjects; thus, improving the FDR for a small sample is extremely helpful for researchers.

For *n* = 15 (per group) and *q* = 0.2, simulated FDR by BLfdr ranges between 0.439 and 0.547, compared to a range between 0.665 and 0.678 by Lfdr. In Figure 2, we see BLfdr (red color) has a better performance across all three correlations for both *q* = 0.20 and 0.30 compared to Lfdr (blue color). In particular, for *n* = 15, better performance of BLfdr with a wider gap between red and blue curves draws our attention.

As sample size increases, FDRs by both methods are reduced greatly. For *n* = 45, FDRs by the two methods are close (but still BLfdr is slightly better than Lfdr), and both converge to the pre-specified level *q*, suggesting that even asymptotic results of BLfdr are slightly better than those of Lfdr. FDRs at *q* = 0.3 are larger than those at *q* = 0.2, which is expected. This simulation also provides a guideline to researchers on determining the appropriate sample size (*n* = 45 per group) when the goal is to control the FDR at the desired level (i.e., *q* = 0.2 or 0.3).

The take-home message of this simulation study is that when the goal is to detect disrupted FC, it is better to incorporate additional information from SC (as BLfdr does, but Lfdr does not) for better controlling the FDR. Determination of appropriate sample size is necessary to effectively control the FDR at a desired level *q*, which this study does. Otherwise, the study will end up with discovering false biomarkers, defeating the purpose of the study.

## 5. Application of BLfdr to LLD data

Let us now revisit the motivational example and analyze the study data. Various assumptions were made while developing a statistical model in Section 2. In this section, first, we show how those assumptions are satisfied with the LLD data and why it is appropriate to take *q* = 0.2 or 0.3. Once the validity of the model is established, we will concentrate on detecting significant disrupted FCs. The details of image acquisition and data processing of the study are described in Tadayonnejad et al. (2014) and Zhao (2014).

As mentioned in the motivating example, the data set has 23 participants; 13 HC and 10 LLD subjects. The adjacency matrix of each of FC and SC has $\left(\begin{array}{c} 87\\ 2 \end{array}\right)=87\times \left(87-1\right)/2=3741$ unique connectivity measures (or links). The SC was measured using an internal Matlab program by counting the number of fiber tracts found by the tractography algorithm connecting each pair of regions. Some SC data have zero values, suggesting no SC between the corresponding brain regions. Since FC data is measured using Pearson's correlation coefficients (*r*), we apply Fisher's *Z* transformation to stabilize the variance and get an approximate normal distribution for the transformed data. Moreover, for SC data, cube-root transformation is applied to get an approximate normal distribution (Bhaumik et al., 2018b).

The *p*-values from *t*-tests to compare mean FC measures between LLD and HC are obtained using a mixed-effects model for all 3741 links. Out of 3,741 hypotheses tests, 281 (7.5%) tests have two-sided *p* ≤ 0.025. This indicates for the existence of a small proportion of alternative links whose FC measurements differ significantly between LLD and HC and some potential false positives.

We ran Markov chains using the prior distribution for model parameters specified before. The acceptance rates for ***α*** and ***γ*** using the multiple-try Metropolis within the Gibbs algorithm were satisfactory. We performed model convergence diagnostics to ensure that it converged to the target posterior distribution and reached the stationarity. We took the posterior medians as the posterior estimates of each parameter. The posterior inference, including medians and 95% credible intervals, are presented in Table 1. The results are based on 9,000 posterior samples combined from three parallel Markov chains, where each chain has 325,000 iterations, with the first 25,000 as burn-in and a thinning interval of 100 after burn-in.

**Table 1:** Posterior inference for model parameters.

**Parameter**	**Median (95% credible interval)**
α_0_	1.120 (0.362, 1.739)
α_1_	−0.018 (−0.548, 0.353)
β	2.214 (1.204, 3.359)
γ_0_	−2.313 (−3.138, −1.484)
γ_1_	−0.692 (−1.680, −0.055)
$\sigma_{0}^{2}$	1.100 (0.829, 1.283)

**Figure 3:**
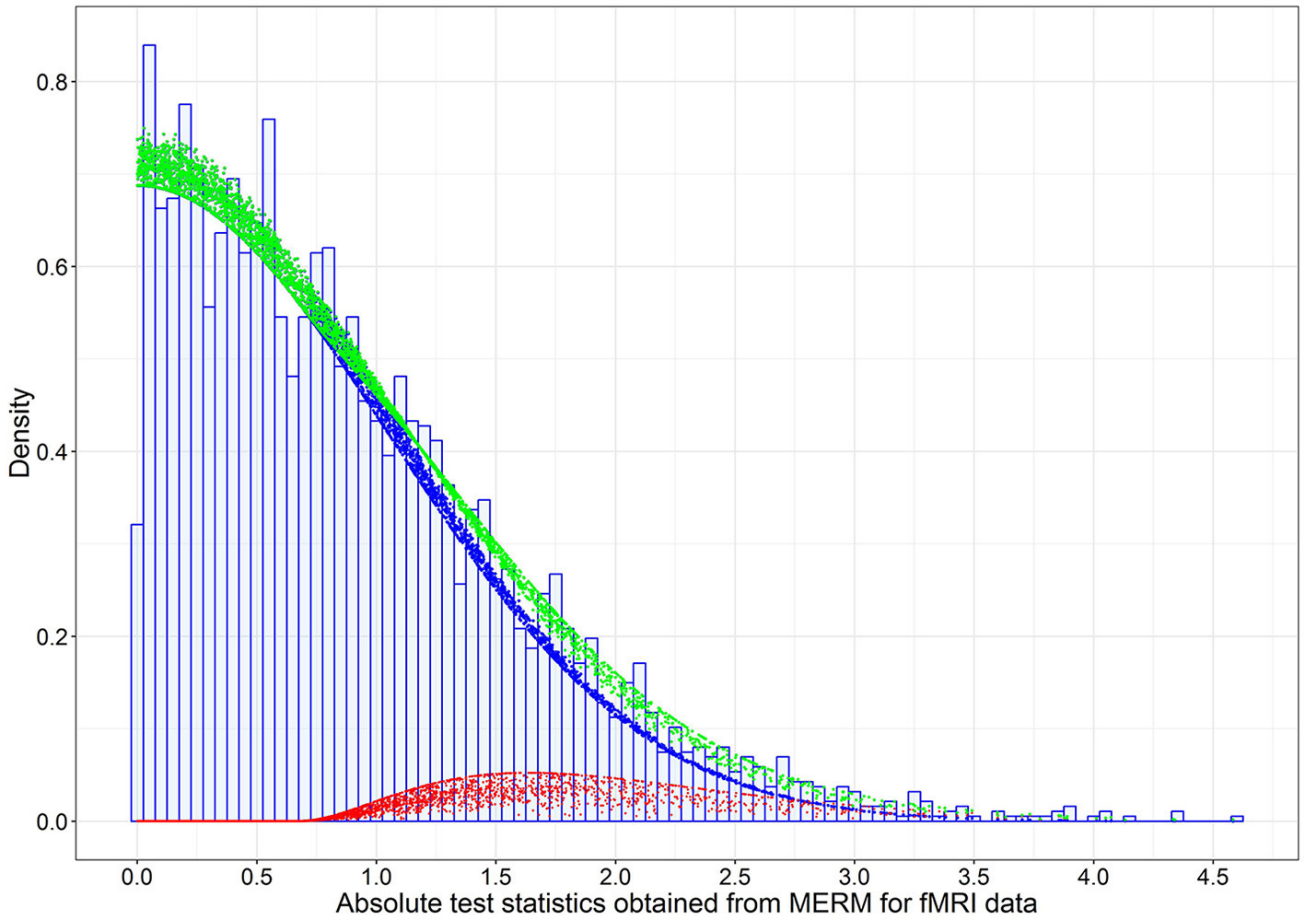
The estimated weighted null, weighted alternative, and mixture densities based on the posterior estimates of model parameters for the 3,741 absolute test statistics obtained from the mixed-effects model on FC data in the LLD study. The blue dots are the weighted null density $\pi_{0}\left(t_{i}^{\left(S\right)}|\hat{\gamma }\right)f_{0}\left(t_{i}^{\left(F\right)}|{\hat{\sigma }}_{0}^{2}\right)$, the red dots are the weighted alternative density $\pi_{1}\left(t_{i}^{\left(S\right)}|\hat{\gamma }\right)f_{1}\left(t_{i}^{\left(F\right)}|\hat{\alpha },\hat{\beta },t_{i}^{\left(S\right)}\right)$, and green dots are the mixture density $\pi_{0}\left(t_{i}^{\left(S\right)}|\hat{\gamma }\right)f_{0}\left(t_{i}^{\left(F\right)}|{\hat{\sigma }}_{0}^{2}\right)+\pi_{1}\left(t_{i}^{\left(S\right)}|\hat{\gamma }\right)f_{1}\left(t_{i}^{\left(F\right)}|\hat{\alpha },\hat{\beta },t_{i}^{\left(S\right)}\right)$, where $\left\{\hat{\alpha },\hat{\gamma },\hat{\beta },{\hat{\sigma }}_{0}^{2}\right\}$ are the posterior estimates (Table 1).

Assuming these FC links were under the null hypothesis, the estimated variance (${\hat{\sigma }}_{0}^{2}$) of the null density is 1.01 using absolute test statistics with *p* > 0.025. This is a reasonable fit for the links under the null hypothesis, while the tail of the histogram for the potential alternative links is fitted using a gamma distribution described in the Section of the Bayesian Mixture Model. Thus, we can obtain a conservative estimate of the proportion of null cases (Storey and Tibshirani, 2003), denoted by ${\hat{\pi }}_{0}$. In Figure 3, the dashed blue line is the weighted null density ${\hat{\pi }}_{0}{\hat{f}}_{0}\left(t_{i}^{\left(F\right)}\right)$, where ${\hat{\pi }}_{0}=\overset{3741}{\underset{i=1}{\sum }}I\left\{p{\text{-value}}_{i}>0.025\right\}/3741\left(1-0.025\right)=3584/\left(3,741\times 0.975\right)=0.983$. The weighted red dots represent the alternative (non-null) density, and the green dots represent mixture density using posterior estimates given in Table 1.

Based on the posterior estimates of the model parameters $\left\{\hat{\alpha },\hat{\gamma },\hat{\beta },{\hat{\sigma }}_{0}^{2}\right\}$, we calculated the posterior probability of each link being null given the observed FC and SC test statistics, ${\hat{BLfdr}}_{i}$, *i* = 1, ..., 3,741, by (9), and then determined which connectivity links to be rejected using the oracle procedure to control the FDR (Sun and Cai, 2007). The Bayesian multimodal Lfdr method detected 21 connectivity links, significant FC difference between the LLD group (*n*_1_ = 10) and HC group (*n*_2_ = 13) at *q* = 0.2 is presented in Tables 2, 3, where 15 connectivity links have increased FC significantly (hyperconnectivities), and six connectivity links have decreased FCs significantly (hypoconnectivities). The primary hub, the right caudal middle frontal (RCMF), is identified by both BLfdr and Lfdr methods.

**Table 2:** FC links identified by BLfdr, Efron's Lfdr, DPM, and TBSS at q = 0.2 for LLD study.

**Region 1^1^**	**Region 2^1^**	**BLfdr^1^^,^^2^**	**Lfdr^1^^,^^2^**	**DPM^1^^,^^2^**	**TBSS^1^^,^^2^**	**ULMM^1^^,^^2^**
**Hypoconnectivities**						
R caudal middle frontal (RCMF)	L rostral middle frontal (LRMF)	X	X	X		
R caudal middle frontal (RCMF)	R pallidum (RP)	X	X			
R caudal middle frontal (RCMF)	R caudal anterior cingulate (RCAC)	X	X			
R caudal middle frontal (RCMF)	R isthmus cingulate (RIC)	X	X			
R caudal middle frontal (RCMF)	R thalamus proper (RTP)	X	X	X		
R caudal middle frontal (RCMF)	R posterior cingulate (RPC)	X		X		
R caudal middle frontal (RCMF)	L isthmus cingulate (LIC)	X	X	X		
R caudal middle frontal (RCMF)	R precentral (RPrC)					X
R accumbens area (RAA)	L isthmus cingulate (LIC)	X	X			
R accumbens area (RAA)	R medial orbitofrontal (RMO)				X	
R accumbens area (RAA)	L caudate (LCau)					X
R posterior cingulate (RPC)	R supramarginal (RS)	X		X		
L posterior cingulate (LPC)	R supramarginal (RS)	X		X		
L posterior cingulate (LPC)	R rostral middle frontal (RRMF)	X	X			
R ventral diencephalon (RVD)	R fusiform (RF)	X	X	X		
R amygdala (RAMY)	R fusiform (RF)					X
R pallidum (RP)	L inferior parietal (LIP)	X	X			
L pallidum (LP)	L inferior parietal (LIP)					X
L pallidum (LP)	L cerebellum (LC)					X
L insula (LI)	L inferior parietal (LIP)					X
L pallidum (LP)	Brain Stem (BS)					X
L inferior parietal(LIP)	L ventral diencephalon(LVD)					X
R amygdala (RAMY)	R ventral diencephalon(RVD)					X
R amygdala (RAMY)	Brain Stem (BS)					X
R superior frontal (RSF)	R caudal middle frontal (RCMF)			X		
R pars triangularis (RPT)	R rostral anterior cingulate (RRAC)	X				
R superior frontal (RSF)	R rostral anterior cingulate (RRAC)				X	
L superior frontal (LSF)	R rostral anterior cingulate (RRAC)				X	
L superior frontal (LSF)	R isthmus cingulate (RIC)		X			
R superior parietal (RSP)	R paracentral (RPA)					X
L superior parietal (LSP)	R superior parietal (RSP)			X		
L superior parietal (LSP)	R pars opercularis (RPO)	X		X		
L superior parietal (LSP)	R supramarginal (RS)			X		
L superior parietal (LSP)	R insula (RI)					X
L superior parietal (LSP)	L lateral occipital(LLO)					X
L superior parietal (LSP)	L parahippocampal (LPH)					X
L isthmus cingulate (LIC)	L parahippocampal (LPH)					X
L precuneus (LPR)	L parahippocampal (LPH)					X
L inferior parietal (LIP)	R posterior cingulate (RPC)			X		
L caudal anteriorc ingulate (LCAC)	R posterior cingulate (RPC)					X
L rostral anterior cingulate (LCAC)	R medial orbitofrontal (RMO)					X
L rostral middle frontal (LRMF)	R medial orbitofrontal (RMO)					X
L lateral orbitofrontal (LLO)	R medial orbitofrontal (RMO)			X		
L lateral orbitofrontal (LLO)	L medial orbitofrontal (LMO)			X		
L frontal pole (LFP)	L medial orbitofrontal (LMO)					X
L precentral (LPrC)	R precentral (RPrC)			X		
L precentral (LPrC)	R postcentral (RPoC)			X		
L postcentral (LPoC)	L precentral (LPrC)			X		
R insula (RI)	R bankssts (RB)					X
R insula (RI)	L lateral occipital (LLO)					X
L insula (LI)	R insula (RI)					X
L insula (LI)	Brain Stem (BS)					X
L insula (LI)	L thalamus proper (LTP)					X
L superior temporal (LST)	L thalamus proper (LTP)					X
R putamen (RPU)	R precuneus (RPR)					X
R putamen (RPU)	R cerebellum (RC)					X
R putamen (RPU)	Brain Stem (BS)					X
Brain Stem (BS)	R temporal pole (RT)					X
Brain Stem (BS)	L superior Temporal (LST)					X

1 L, left; R, right; LLD, late-life depression; HC, health control; Blfdr, Bayesian Lfdr model; Lfdr, Efron's local discovery rate model; DPM, Dirichlet process mixture; TBSS, tract-based spatial statistics; ULMM, univariate linear mixed-effects model for SC links.

2 The connectivity links identified by each method are indicated using X.

**Table 3:** FC links identified by BLfdr, Efron's Lfdr, DPM, and TBSS at q = 0.2 for LLD study.

**Region 1^1^**	**Region 2^1^**	**BLfdr^1^^,^^2^**	**Lfdr^1^^,^^2^**	**DPM^1^^,^^2^**	**TBSS^1^^,^^2^**	**ULMM^1^^,^^2^**
**Hypoconnectivities**						
R caudate (RCau)	R cuneus (RCun)			X		
R caudate (RCau)	L cuneus (LCun)	X				
L fusiform (LF)	L pars triangularis (LPT)	X				
L fusiform (LF)	L supramarginal (LS)	X	X	X		
L entorhinal (LE)	L supramarginal (LS)	X				
L thalamus proper (LTP)	L posterior cingulate (LPC)	X				
L ventral diencephalon (LVD)	R caudal anterior cingulate (RCAC)	X				

1 L, left; R, right; LLD, late-life depression; HC, health control; Blfdr, Bayesian Lfdr model; Lfdr, Efron's local discovery rate model; DPM, Dirichlet process mixture; TBSS, tract-based spatial statistics; ULMM, univariate linear mixed-effects model for SC links.

2 The connectivity links identified by each method are indicated using X.

To assess the consistency of our results between the BLfdr and Lfdr methods, we compared the significant FC links identified using these two methods. Using Lfdr by R locfdr package, we get the central matching estimates of the null distribution as $f_{0}\sim Normal\left({\hat{\mu }}_{0}=0.038,{\hat{\sigma }}_{0}^{2}=1.250\right)$ and the null proportion as $\hat{p}=0.988$. This large value of $\hat{p}$ justifies using the FDR *q* = 0.2 or 0.3. The estimated null density has a variance of 1.250, which is larger than 1.100 using BLfdr. The Lfdr method detects 12 significant FC links at *q* = 0.2, among which 11 links overlap with those identified by BLfdr. Matching with the simulated results, we would like to say that borrowing strength from SC, BLfdr controls the FDR better than Lfdr. Further, to compare our results, we used a semi-parametric Bayesian approach utilizing a nonparametric Dirichlet process mixture model (DPM) (Ghosal, 2019) and Tract-Based Spatial Statistics (TBSS) as discussed in Tadayonnejad et al. (2014). It should be noted that the primary purpose of developing the TBSS was not for classification. Significant FC links detected by various methods are presented in Tables 2, 3. Significant SC links detected by the Univariate Linear Mixed-Effects Model (ULMM) at *q* level of 0.3 are presented in Tables 2, 3.

### 5.1. Accuracy in classification

We examine the performance of our approach while classifying subjects with selected links presented in Tables 2, 3. Random Forest, an embedded learning regression method with a leave-one-out cross-validation strategy, is used to classify subjects with selected features presented in Tables 2, 3. Performance of each approach is measured by sensitivity, specificity, accuracy, and area under the curve (AUC), and results are presented in Table 4. BLfdr has a very high sensitivity (89.05%), specificity (87.63%), accuracy (88.54%), and AUC (98.46%) and performs much better compared to all other methods. One reason for the better performance of BLfdr is the additional strength it borrows from SC. The high accuracy rate of classification of BLfdr encourages us to use it for prediction of early prevention.

**Table 4:** Classification performance of Lfdr, BLfdr, DPM, and TBSS at q = 0.2 for LLD study.

**Model**	**Accuracy**	**Sensitivity**	**Specificity**	**AUC**
BLfdr	0.8854	0.8905	0.8763	0.9846
Lfdr	0.7964	0.8668	0.7043	0.9000
DPM	0.7905	0.8027	0.7705	0.9154
TBSS	0.6996	0.8074	0.5560	0.6731

## 6. Model comparison with multimodality

Multimodal neuroimaging data are used to better understand the neurological condition of brain with diseases. Neuroimaging studies with multimodality mainly focus on correlations of modalities or detection of the main components of such correlations. Sui et al. (2013) employed the independent component analysis to estimate the number of independent components using three modalities such as fMRI, DTI, and structural MRI while studying the abnormal structure underlying schizophrenia relative to healthy controls. Zhao (2014) used a bivariate linear mixed-effects model (BLMM) to detect disrupted links of LLD compared to HC. Honey et al. (2009) investigated the correlation between FC (using resting-state fMRI) with SC (using DTI).

Our proposed Bayesian model, i.e., BLfdr, utilizes SC as an auxiliary information to model FC. To enable comparisons, we used the BLMM to model FC and SC jointly. Controlling the FDR at a level of 0.2, we identified 40 significant links for both FC and SC, only two links (left ventral diencephalon (LVD)—right caudal anterior cingulate (RCAC) and left thalamus proper (LTP)—left posterior cingulate (LPC) overlapped with those detected by the BLfdr model. However, considering brain regions as a whole, we found that 15 regions of interest (ROIs) identified by the BLMM overlapped with those detected by the BLfdr model (refer to Table 5). Moreover, BLMM requires subjective decisions regarding the covariance matrix between SC and FC, and it takes a long computational time due to the setting of bivariate outcomes. In contrast, our proposed Bayesian approach is straightforward and properly leverages multimodal information to detect disrupted functional connectivity.

**Table 5:** Fifteen ROIs detected by BLMM that overlapped with those found by BLfdr.

**Network**	**ROIs**
Default mode network (DMN)	L inferior parietal (LIP)
	L/R posterior cingulate (LPC/RPC)
	L/R thalamus proper (LTP/RTP)
Salience network (SN)	R caudal anterior cingulate (RCAC)
Central executive network (CEN)	L superior parietal (LSP)
	R caudal middle frontal (RCMF)
	R rostral middle frontal (RRMF)
Visual network/Fusiform face area (VN/FFA)	L cuneus (LCun)
	L fusiform (LF)
Reward network (RN)	L ventral diencephalon(LVD)
	R accumbens area (RAA)
	R caudate (RCau)
	R pallidum (RP)

### 6.1. Model-based analysis results

The traditional method for comparing LLD with HC using FC or SC data typically involves (i) *t*-tests that ignore between-link correlations and (ii) Hotelling's T-square tests. In addition, we compared the two groups by (iii) Fisher's *Z* transformation on Pearson correlation of SC and FC. Using (i), we observed 59 significant FCs and 49 significant SCs; (ii) produced 55 significant links for joint FC and SC, and (iii) identified 72 significant links for FC-SC correlations. However, none of these analyses remained significant after controlling for the multiplicity problem and type I error rate using an FDR level of 0.30. We observed almost no overlapping results between FC and SC in (i); and between (i) and (iii). As expected, (i) and (ii) share about 50% of common significant links.

Using the univariate linear mixed-effects model (ULMM) and controlling the FDR level of 0.3, we detected 28 significant FCs and 31 significant SCs. SC results of ULMM are presented in the last column of Tables 2, 3. The bivariate mixed-effects model detected 40 and 90 significant links when the FDR level was controlled at 0.2 and 0.3, respectively. An interesting finding of this analysis is that (a) the proportion of false discoveries (FDP) for FC with within-subject independence was 0.52, and with within-subject covariance was 0.06, (b) the FDP for SC with within-subject independence was 0.36, and with within-subject covariance was 0.04. These results suggest that when an appropriate covariance structure is implemented, the FDP is close to the desired value of 0.05. However, when the correlation structure was ignored, FDP exceeded the desired level of 0.50.

One possible explanation for these findings is that when tests are correlated, an appropriate dependency structure must be implemented in the computation of the FDP. Highly correlated hypotheses that favor the alternative are likely to be rejected together, while hypotheses that favor the null are likely to be retained together because of their dependency. If the FDP value is small, it indicates that the number of false discoveries is not large, and any hypothesis that is not rejected is likely not to be significant. However, under the assumption of an uncorrelated structure, if the number of rejections remains the same, the rejected hypotheses are more likely to be randomly selected from all hypotheses, leading to an inflated FDP. These results suggest that the underlying correlation structure plays a vital role in controlling the FDP when dealing with multiple testing problem, and ignoring such correlations increases the likelihood of false discoveries.

**Figure 4:**
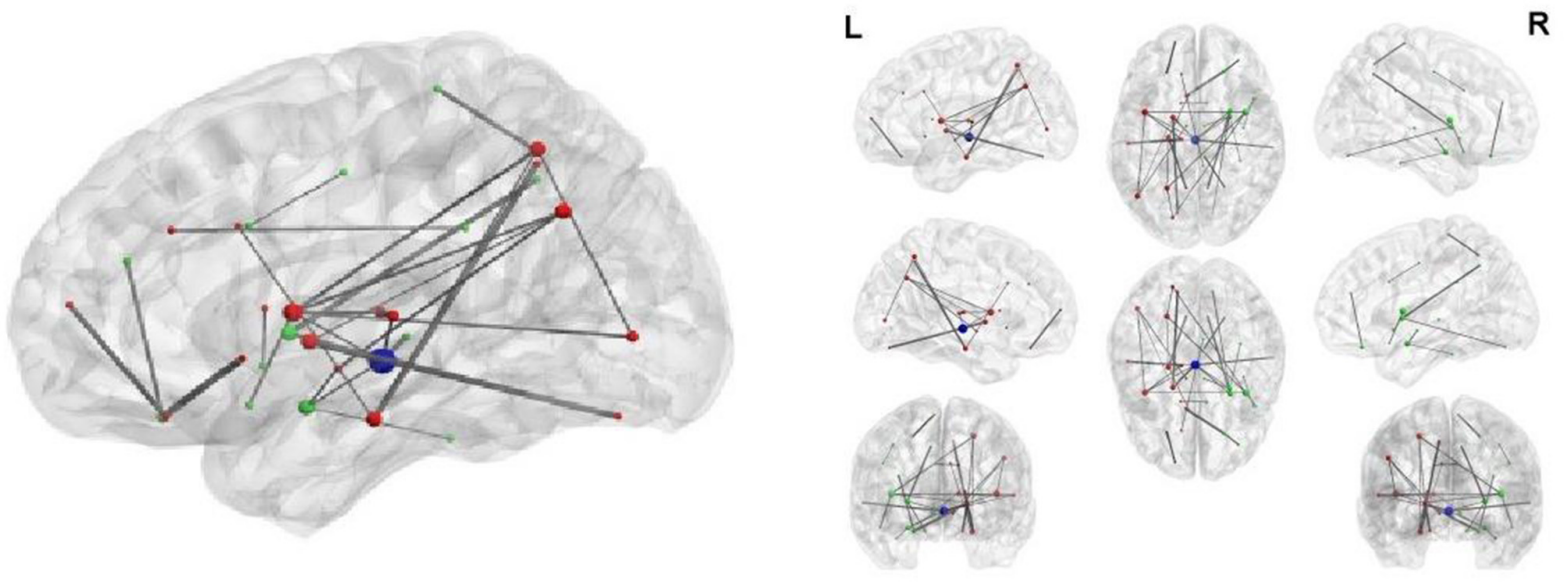
Thirty-one structural connectivity with the significant between-group difference.

Using a bivariate mixed-effects model with FC and SC, we identified the left inferior parietal as the hub of impaired regions. Bayesian results are comparable with those described above by the bivariate mixed-effects model. However, SC links detected by ULMM using SC are not comparable with FC links detected by the bivariate mixed-effects model or BLfdr using FC and SC. Figure 4 displays the 31 disrupted SC links detected by the ULMM.

## 7. Neurophysiological importance of our discovery

**Figure 5:**
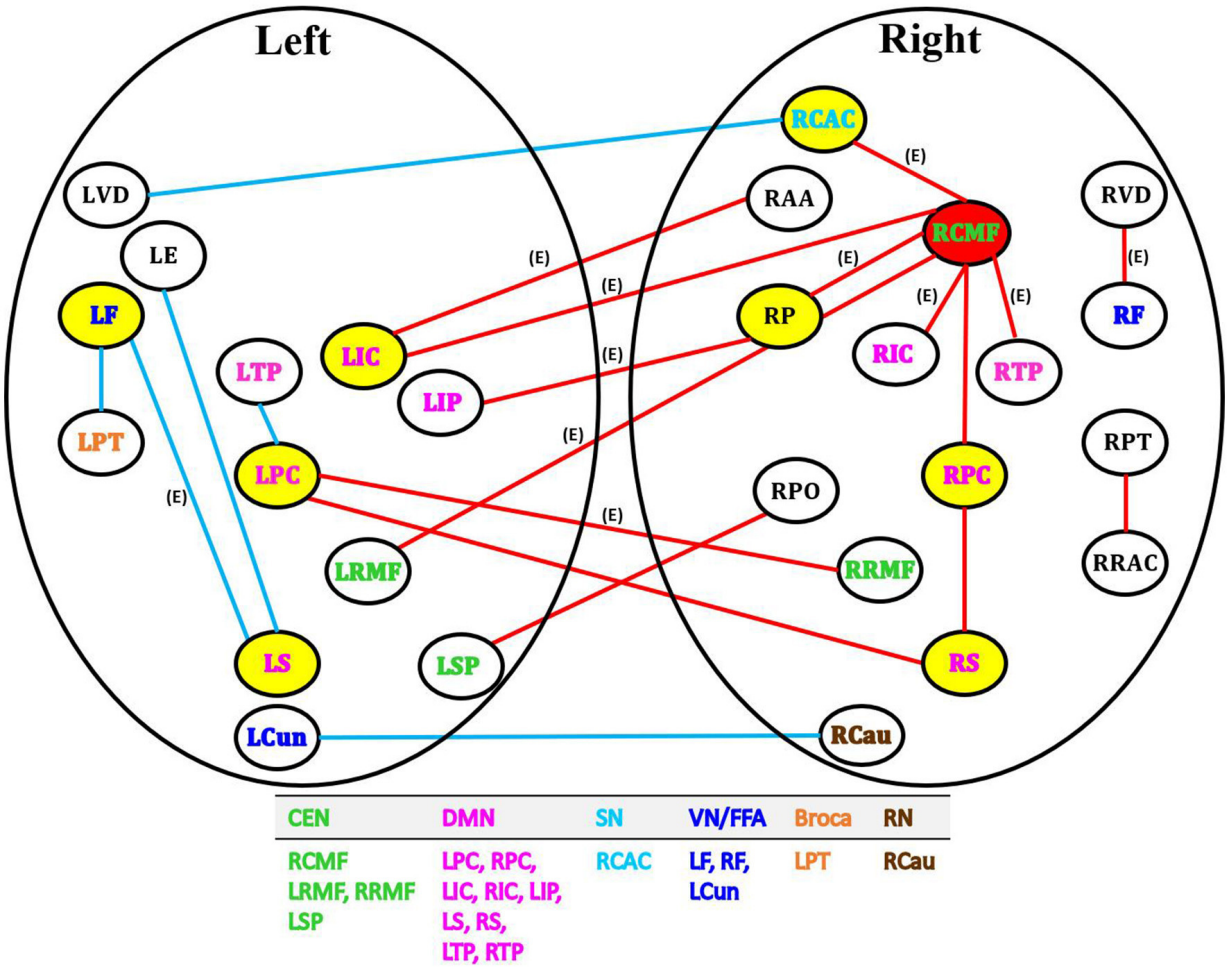
Network analysis of 21 FC links by BLfdr at FDR level of 0.2 in cortical and subcortical gray matter regions in the left and right hemispheres of brain. Red lines indicate an increase in FC (hyperconnectivity), and blue lines indicate a decrease in FC (hypoconnectivity). Red circle denotes the primary hub, and yellow circles represent secondary hubs. Eleven FC links, also identified by Efron's Lfdr method, are shown using (E). CEN, central executive network; DMN, default mode network; SN, salience network; VN/FFA, visual network/fusiform face area; Broca, Broca's area; RN, reward network.

BLfdr with *q* = 0.2 detects 21 significantly disrupted links on cortical and subcortical gray matter regions in the left and right hemispheres of the brain. Figure 5 displays all FC connectivity links significantly different between the LLD and HC groups. The primary hub—the right caudal middle frontal, also known as the right dorsolateral prefrontal cortex (dlPFC), is identified. In LLD patients, we find significantly increased FCs in seven other brain regions, including the bilateral isthmus cingulate (LIC/RIC), left rostral middle frontal (LRMF), right thalamus proper (RTP), right posterior cingulate (RPC), right pallidum (RP), and right caudal anterior cingulate (RCAC). The dlPFC area has been determined as the critical neural substrate for MDD. This area controls cognitive and complex mental processes, including emotion modulation, selective attention, and working memory (Hamilton et al., 2012). Some literature indicates that healthy subjects showed bilateral activation during a working memory task. In contrast, depressed patients exhibited asymmetric activity, with the left dlPFC showing an increased activation (Perrin et al., 2012). In 2008, the US Food and Drug Administration (FDA) approved a treatment for MDD. This treatment (FDA approval K061053) utilizes the repetitive transcranial magnetic stimulation (rTMS) system, which delivers transcranial repetitive pulsed magnetic fields of sufficient magnitude that induces neural action potentials in the left dlPFC area. Additionally, randomized clinical trials for depressed subjects established the clinical effectiveness of rTMS on the left dlPFC (Blumberger et al., 2018).

Our analysis shows a strong evidence of the laterization of right dlPFC with significant increased FCs; i.e., a specific and distinctive FC activation path via the right dlPFC was found in LLD patients. The right dlPFC with an increased FC also served as a critical hub involved in cognition impairment and LLD pain. Ihara et al. (2019) found altered FCs in the right dlPFC of patients with chronic neck pain at high risk for depression. Chronic pain is one of the most common comorbid conditions among patients with LLD (Aziz and Steffens, 2013). Thus, the right dlPFC with an increased FC is the crucial region for cognition impairment and pain for LLD.

We present the remaining results of our study in Table 6, where detected connectivity is given in Column 2, and the corresponding network(s) to which it belongs to is stated in Column 1. The functional activity of detected links and corresponding references are presented in Column 3.

**Table 6:** FC links within CEN, SN, RN, DMN, Broca, and VN/FFA identified using BLfdr for LLD study.

**Network**	**[+/−] ROIs^1^**	**Function of the ROIs/Network**
Central executive network (CEN)	RCMF (CEN) vs. LIC/RIC (DMN)	(1) CEN controls cognitive and mental processes, including emotion modulation, selective attention, and working memory. (2) RCMF is also called dlPFC, which served as the key neural substrates for MDD (Hamilton et al., 2012). (3) Depressed patients exhibit asymmetric activity in dlPFC (Perrin et al., 2012). The left dlPFC shows increased activation. The right dlPFC is a critical hub of the altered FCs in chronic net pain patients at high risk of experiencing depression (Aziz and Steffens, 2013; Ihara et al., 2019). (4) The left dlPFC has been the target site of repetitive transcranial magnetic stimulation (rTMS) as a treatment for medication-resistant MDD (refer to FDA approval K061053; Blumberger et al., 2018).
	RCMF (CEN) vs. LRMF/RRMF (DMN)	(1) RRMF is a converging site of the dorsal and ventral attention networks by playing a role in the reorientation of attention.
	LSP (CEN) vs. RPO (IFG)	(1) RPO belongs to the right inferior frontal gyrus (IFG), which is associated with cognitive functions, including speech, attention, motor inhibition, imagery. (2) Hyperconnectivity of RPO was observed in adolescents with MDD (Tang et al., 2018).
Salience network (SN)	RCAC (SN) vs. RCMF (dlPFC)	(1) SN works in detecting, filtering, and integrating salient external stimuli with internal states to orchestrate brain network dynamics in the service of goal-directed behaviors and motivated behaviors. (2) SN plays a mediating role in switching between activation and deactivation of internally directed cognition of DMN and externally directed cognition of CEN. (3) SN is associated with MDD/LLD (Yuen et al., 2014). (4) The caudal anterior cingulate (CAC, aka. dACC) and anterior insula are two major cortical structures of the SN.
Reward network (RN)	[-] RCau (RN) vs. LCun (VN/FEA)	(1) RN includes reinforcement learning, action monitoring, novelty processing, learning, decision making, and addiction. (2) RCau is a key region with enhanced neural response to positive emotions in MDD (Keren et al., 2018).
Default mode network (DMN)	RPC (DMN) vs. RCMF (CEN) and [+] LPC (DMN) vs. RRMF (CEN)	(1) DMN, the default mode of brain functions for internally directed self-referential cognition activated at rest and deactivated during cognitive and mental tasks requiring attention and response. (2) DMN is associated with MDD (Kaiser et al., 2015). (3) The posterior cingulate cortex (PCC)—is considered the posterior hub of the DMN, controlling emotion, cognition, awareness, arousal, and regulatory modulation. PCC exhibits higher activities when the brain is at rest. (4) PCC is associated with MDD (Zhou et al., 2010; Zhao et al., 2019) and LLD (Alexopoulos et al., 2012).
	[-] LS (DMN) vs. LF (VN/FFA) and [+] RS (DMN) vs. LPC/RPC (DMN)	(1) Supramarginal gyrus (LS/RS) is part of the inferior parietal lobe that functions in sensory-motor and cognitive domains. (2) Inferior parietal lobe is associated with depression symptoms (Roh et al., 2020).
	[-] LTP (DMN) vs. LPC (DMN) and [+] RTP (DMN) vs. RCMF (CEN)	(1) The bilateral thalamus proper (LTP/RTP), also called Dorsal Thalamus (DT). (2) DT is a hub for relaying sensory and motor signals from the senses to the cerebral cortex and receiving feedback from the cortex. DT plays a mediator role in-between cortico-cortical communication processing (Sherman and Guillery, 2002). (3) DT is associated with MDD (Greicius et al., 2007).
Broca's area (Broca)	[-] LPT (Broca) vs. LF (VN/FFA)	(1) Broca is essential for the speech process by integrating and coordinating information. (2) Pars Triangularis (PT) is considered a hub of the language-control network (Elmer, 2016). (3) LPT has been observed to activate semantic processing (Friederici et al., 2000).
Visual Network / Fusiform Face Area (VN/FFA)	RF (VN/FFA) vs. RVD	(1) The bilateral fusiform gyrus (FG) involves in visual cognition, especially facial cognition. (2) The antidepressants have improved the neural response to positive emotion in the LF and the right dlPFC (Ma, 2015). (3) RVD is associated with LLD (Lebedeva et al., 2017).

^1^[+] indicates increased FCs and [-] indicates decreased FCs.

Thus, our findings match most of those in the literature. Our results of increased FC in the bilateral PCC left IPL, and left supramarginal (LS) within the DMN of LLD patients, together with those in the literature, suggest that interventions targeting to decrease FCs within the DMN may have beneficial clinical effects for LLD patients. Menon (2015) suggested that the caudal anterior cingulate (CAC) and anterior insula are two major cortical structures of the SN. Moreover, electroconvulsive therapy can help reduce FCs between the right anterior cingulate cortex and the right dlPFC in patients with severe depression (Perrin et al., 2012). Our results consistently show increased FC between the right dACC within the SN, the right dlPFC within the CEN, and the right PCC within the DMN, across the major large-scale neurocognitive brain networks, including the DMN, CEN, and SN of LLD patients. These critical brain networks may contribute to a deterioration of memory and cognitive functions in elderly patients with LLD.

## 8. Conclusions and discussions

Based on the maximum number of disrupted links and their cognitive importance, we would like to declare the right caudal middle frontal (RCMF), also known as dlPFC, as a possible biomarker. However, it needs larger studies to confirm this result. The long-term goal of this article is to treat medicine-resistant LLD patients who need alternative treatments. Exploring the association of RCMF with the central executive network's function that controls cognitive and complex mental processes, including emotion modulation and selective attention working memory, neuropsychotherapies (e.g., cognitive behavioral therapy) can be developed. Pinpointing the RCMF in the broad area of the central executive network is extremely helpful to use as a target for applying neurotherapeutic intervention (e.g., transcranial magnetic stimulus, TMS) to get optimal stimulus results. The high accuracy rate of classification encourages us to use RCMF to predict prospective subjects at high risk for early prevention. The team is currently investigating the cognitive gain from applying neurotherapeutic intervention on RCMF, the detected biomarker. Our approach can be extended to traumatic brain injury studies to select the target for TMS or PTSD study for treatment selection. It is worth to extend our methodology for longitudinal neuroimaging studies when detected biomarker needed to be consistent over time.

## Data availability statement

The original contributions presented in the study are included in the article/Supplementary material, further inquiries can be directed to the corresponding author.

## Ethics statement

The studies involving human participants were reviewed and approved by UIC Institutional Review Board. The patients/participants provided their written informed consent to participate in this study.

## Author contributions

DB and YW conceived the novel model. DB, YW, and P-SY contributed equally to revising the initial draft. YW conducted the initial analyses and P-SY assisted with the classification performance evaluation. OA provided the LLD data and helped with the interpretation of neurophysiological discovery results. All authors contributed to the article and approved the submitted version.
