# Supplementary material for: Development of a Bayesian multimodal model to detect biomarkers in neuroimaging studies
Source: Front Neuroimaging. 2023 May 24;2:1147508. doi: 10.3389/fnimg.2023.1147508 (PMC10406277; doi:10.3389/fnimg.2023.1147508)
Supplement: Supplementary file 1 [file Data_Sheet_1.pdf]

frontiers

# Development of a Bayesian Multimodal Model to Detect Biomarkers in Neuroimaging Studies

**Dulal K. Bhaumik** [1,3], **Yue Wang** [2], **Pei-Shan Yen** [1,*], **and Olusola A. Ajilore** [3]

[1] *Division of Epidemiology and Biostatistics, University of Illinois at Chicago, Chicago, IL, United States*
[2] *Biostatistics, Incyte Corporation, Wilmington, DE, United States*
[3] *Department of Psychiatry, University of Illinois at Chicago, Chicago, IL, United States*

Correspondence*:
Pei-Shan Yen
pyen2@uic.edu

## 1 APPENDIX

### 1.1 Simulation Algorithm

This simulation study is designed following a data structure similar to that of the motivating example. To account for the potential correlation between FC and SC, we use a bivariate mixed-effects model with heteroscedastic errors (Zhao, 2014). FC and SC data are jointly simulated considering within one sigma deviation of parameters estimated from the mixed-effects model using the LLD neuroimaging data.

By mimicking the sample size of the LLD study with 10 in the LLD group and 13 in the HC group, we consider small to moderate sample sizes for both LLD and HC group: $n = 15, 25, 35$ and $45$ per group, with FC and SC measurements taken from 87 ROIs for each participant. The total number of connectivity links measured is $m = 87 \times 86/2 = 3741$.

Let $H_{0,i}^{(F)}$ and $H_{0,i}^{(S)}$ be the null hypothesis that the $i^{th}$ link has no difference between LLD group and HC group in FC and SC, respectively. For simplicity, we refer to the connectivity links under the null hypothesis as null connectivity links and the connectivity links under the alternative hypothesis as alternative connectivity links. The $3741$ connectivity links can be classified into four-link classes based on $H_{0,i}^{(F)}$ and $H_{0,i}^{(S)}$ (see Table 1).

**Table 1.** Classification of connectivity links in simulation study

| $H_{0,i}^{(F)}$ | $H_{0,i}^{(S)}$ | Link Class Description |
|---|---|---|
| False | False | alternative in both FC and SC |
| False | True | alternative in FC, null in S |
| True | False | null in FC, alternative in SC |
| True | True | null in both FC and SC |

Following the LLD neuroimaging dataset, we assume that

1. The proportion of connectivity links that are alternative in both FC and SC is $1\%$,
2. The proportion of connectivity links that are alternative in FC and null in SC is $1\%$,
3. The proportion of connectivity links that are null in FC and alternative in SC is $1\%$,
4. The proportion of connectivity links that are null in both FC and SC is $97\%$.

So the total proportion of alternative links in FC is $2\%$, consistent with the assumption used in the simulation study based on the same LLD neuroimaging data (Song, 2016; Bhaumik et al., 2018). Next, we assume, without a loss of generality, that in each simulated dataset the $3741$ connectivity links are set in the following order:

1. The first 37 (1%) connectivity links are alternatives in both FC and SC,
2. The following 37 (1%) connectivity links are alternative in FC and null in SC,
3. The next 37 (1%) connectivity links are null in FC and alternative in SC,
4. The rest of 3630 (97%) connectivity links are null in both FC and SC.

Let $\delta^{(F)}$ and $\delta^{(S)}$ denote the true differences between the LLD group and HC group for the alternative connectivity links in FC and SC, respectively. We assume $\delta^{(F)}$ follows a uniform distribution $U(0.055, 0.095)$ with a mean of $0.075$, and $\delta^{(S)}$ follows another uniform distribution $U(0.15, 0.35)$ with a mean of $0.25$ considered as moderate differences. Let $y_{ij}^{(F)}$ and $y_{ij}^{(S)}$ be FC and SC observations, respectively, for the $i^{th}$ connectivity link ($i = 1, \cdots, m$) of the $j^{th}$ subject ($j = 1, \cdots, N$, where $N$ is the total number of subjects and $N = 2n$), $y_{ij}^{(F)}$ and $y_{ij}^{(S)}$ are generated using the bivariate mixed-effects model with the following settings,

$$\boldsymbol{y}_{ij} = \begin{bmatrix} y_{ij}^{(F)} & y_{ij}^{(S)} \end{bmatrix}^T, \tag{1}$$

In the bivariate mixed-effects model, $\boldsymbol{y}_{ij}$ can be partitioned into fixed effects $\boldsymbol{\beta}_{0i}$ and $\boldsymbol{\beta}_{1i}$, random subject effects $\boldsymbol{\gamma}_j$ and random errors $\boldsymbol{\epsilon}_{ij}$,

$$\boldsymbol{y}_{ij} = \boldsymbol{\beta}_{0i} \times (1 - Grp_j) + \boldsymbol{\beta}_{1i} \times Grp_j + \boldsymbol{\gamma}_j + \boldsymbol{\epsilon}_{ij}, \tag{2}$$

where

- $Grp_j$ is the indicator variable for which group the subject is from, HC group if $Grp_j = 0$, or LLD group if $Grp_j = 1$.
- $\boldsymbol{\beta}_{0i}$ and $\boldsymbol{\beta}_{1i}$ are the vectors of fixed FC and SC effects for the $i^{th}$ connectivity link in HC group and LLD group, respectively.
- $\boldsymbol{\gamma}_j = \begin{bmatrix} \gamma_j^{(F)} & \gamma_j^{(S)} \end{bmatrix}^T$ is the vector of random subject effects for FC and SC in the $j^{th}$ subject.
- $\boldsymbol{\epsilon}_{ij} = \begin{bmatrix} \epsilon_{ij}^{(F)} & \epsilon_{ij}^{(S)} \end{bmatrix}^T$ is the vector of random errors for FC and SC of the $i^{th}$ connectivity link in the $j^{th}$ subject.
- $\boldsymbol{\gamma}_j$ and $\boldsymbol{\epsilon}_{ij}$ are independent of each other.

For fixed effects $\boldsymbol{\beta}_{0i}$ and $\boldsymbol{\beta}_{1i}$, the vectors of fixed FC and SC effects for the $i^{th}$ connectivity link in HC group and LLD group are,

$$\boldsymbol{\beta}_{0i} = \begin{bmatrix} \beta_{0i}^{(F)} & \beta_{0i}^{(S)} \end{bmatrix}^T,$$
$$\boldsymbol{\beta}_{1i} = \begin{bmatrix} \beta_{1i}^{(F)} & \beta_{1i}^{(S)} \end{bmatrix}^T,$$

Let $\bar{\hat{\beta}}_{0+}^{(F)}$ and $\bar{\hat{\beta}}_{0+}^{(S)}$ be the average of the fixed effect estimates overall 3741 connectivity links in HC group for FC and SC from mixed-effects model using the LLD neuroimaging data, respectively, $\bar{\hat{\beta}}_{0+}^{(F)} = \sum_{i=1}^{3741} \hat{\beta}_{0i}^{(F)}/3741 = 0.138$, $\bar{\hat{\beta}}_{0+}^{(S)} = \sum_{i=1}^{3741} \hat{\beta}_{0i}^{(S)}/3741 = 1.099$. We assume,

- For HC group: $\beta_{0i}^{(F)} = \bar{\hat{\beta}}_{0+}^{(F)} = 0.138,\ \beta_{0i}^{(S)} = \bar{\hat{\beta}}_{0+}^{(S)} = 1.099,\ i = 1, ..., 3741$. That is,

$$\boldsymbol{\beta}_0^{(F)} = (\underbrace{0.138, \cdots, 0.138}_{3741})^T,$$

$$\boldsymbol{\beta}_0^{(S)} = (\underbrace{1.099, \cdots, 1.099}_{3741})^T,$$

- For LLD group:

$$\beta_{1i}^{(F)} = \begin{cases} \bar{\hat{\beta}}_{0+}^{(F)} = 0.138 & \text{for null links in FC ,} \\ \bar{\hat{\beta}}_{0+}^{(F)} + \delta^{(F)} = 0.213 & \text{for mean alternative links in FC,} \end{cases}$$

$$\beta_{1i}^{(S)} = \begin{cases} \bar{\hat{\beta}}_{0+}^{(S)} = 1.099 & \text{for null links in SC,} \\ \bar{\hat{\beta}}_{0+}^{(S)} + \delta^{(S)} = 1.349 & \text{for mean alternative links in SC,} \end{cases}$$

That is for mean $\boldsymbol{\beta}_1^{(F)}$, and mean $\boldsymbol{\beta}_1^{(S)}$, values are respectively

$$\boldsymbol{\beta}_1^{(F)} = (\underbrace{0.213, \cdots, 0.213}_{74}, \underbrace{0.138, \cdots, 0.138}_{3667})^T,$$

$$\boldsymbol{\beta}_1^{(S)} = (\underbrace{1.349, \cdots, 1.349}_{37}, \underbrace{1.099, \cdots, 1.099}_{37}, \underbrace{1.349, \cdots, 1.349}_{37}, \underbrace{1.099, \cdots, 1.099}_{3630})^T,$$

Note that values of $\boldsymbol{\beta}_1^{(F)}$, and $\boldsymbol{\beta}_1^{(S)}$ will be changing as $\delta^{(F)}$ and $\delta^{(S)}$ will be drawn from their respective uniform distributions. For random subject effects $\boldsymbol{\gamma}_j$, vector of random subject effects for FC and SC in the $j^{th}$ subject is $\boldsymbol{\gamma}_j = \begin{bmatrix} \gamma_j^{(F)} & \gamma_j^{(S)} \end{bmatrix}^T$. Assume $\gamma_j^{(F)}$ and $\gamma_j^{(S)}$ are independent of each other,

$$\boldsymbol{\gamma_j} = \begin{bmatrix} \gamma_j^{(F)} & \gamma_j^{(S)} \end{bmatrix}^T \sim N(\mathbf{0}, \boldsymbol{\Sigma}_\gamma),$$

$$\boldsymbol{\Sigma}_\gamma = \begin{bmatrix} \sigma_\gamma^{2(F)} & 0 \\ 0 & \sigma_\gamma^{2(S)} \end{bmatrix},$$

where $\sigma_\gamma^{2(F)} = 0.004$ and $\sigma_\gamma^{2(S)} = 0.108$, estimated from mixed-effects model using the LLD neuroimaging data.

For random errors $\boldsymbol{\epsilon}_{ij}$, the vector of random errors for FC and SC of the $i^{th}$ connectivity link in the $j^{th}$ subject is $\boldsymbol{\epsilon}_{ij} = \begin{bmatrix} \epsilon_{ij}^{(F)} & \epsilon_{ij}^{(S)} \end{bmatrix}^T$ and we assume,

- For HC group:

$$\boldsymbol{\epsilon_{ij}} \sim N(\mathbf{0}, \boldsymbol{\Sigma}_{\epsilon,0}),$$

$$\boldsymbol{\Sigma}_{\epsilon,0} = \begin{bmatrix} \sigma_{\{\epsilon,0i\}}^{2(F)} & \rho_{\epsilon,0}\sigma_{\{\epsilon,0i\}}^{(F)}\sigma_{\{\epsilon,0i\}}^{(S)} \\ \rho_{\epsilon,0}\sigma_{\{\epsilon,0i\}}^{(F)}\sigma_{\{\epsilon,0i\}}^{(S)} & \sigma_{\{\epsilon,0i\}}^{2(S)} \end{bmatrix},$$

$$\sigma_{\{\epsilon,0i\}}^{2(F)} \sim \Gamma\left(\alpha_0^{(F)} = 4.34, \beta_0^{(F)} = 120.67\right),$$

$$\sigma_{\{\epsilon,0i\}}^{2(S)} \sim \Gamma\left(\alpha_0^{(S)} = 0.71, \beta_0^{(S)} = 0.64\right),$$

where the shape and rate parameters for the variances are estimated by fitting a gamma distribution to the variance estimates of all 3741 links in the HC group for FC and SC separately, $\rho_{\epsilon,0}$ is the correlation

between the random FC and SC errors in subjects from HC group and we set $\rho_{\epsilon,0} = 0.1$, estimated from the correlation between the variance estimates of all links in HC group for FC and SC, from the mixed-effects model in the lLD neuroimaging study.

- For LLD group:

$$
\begin{aligned}
\boldsymbol{\epsilon_{ij}} &\sim N\left(\mathbf{0}, \boldsymbol{\Sigma}_{\epsilon,1}\right), \\
\boldsymbol{\Sigma}_{\epsilon,1} &= \begin{bmatrix} \sigma^{2(F)}_{\{\epsilon,1i\}} & \rho_{\epsilon,1}\sigma^{(F)}_{\{\epsilon,1i\}}\sigma^{(S)}_{\{\epsilon,1i\}} \\ \rho_{\epsilon,1}\sigma^{(F)}_{\{\epsilon,1i\}}\sigma^{(S)}_{\{\epsilon,1i\}} & \sigma^{2(S)}_{\{\epsilon,1i\}} \end{bmatrix}, \\
\sigma^{2(F)}_{\{\epsilon,1i\}} &\sim \Gamma\left(\alpha_1^{(F)} = 3.49, \beta_1^{(F)} = 89.47\right), \\
\sigma^{2(S)}_{\{\epsilon,1i\}} &\sim \Gamma\left(\alpha_1^{(S)} = 0.66, \beta_1^{(S)} = 0.69\right),
\end{aligned}
$$

where the shape and rate parameters for variances are estimated by fitting a gamma distribution to the variance estimates of all $3741$ links in the LLD group for FC and SC separately from the mixed-effects model using the LLD neuroimaging study.

For both HC and LLD scenarios, corresponding gammas have strong variability; hence no more variability on parameters is imposed. With respect to the correlation coefficient between the random FC and SC errors in subjects from the LLD group (denoted by $\rho_{\epsilon,1}$), we assume that (i) weak correlation (i.e., $\rho_{\epsilon,1} = 0.1$) for null connectivity FC links; (ii) varying scenarios in terms of $\rho_{\epsilon,1}$ for alternative connectivity FC links including weak, mild and strong correlations between random FC and SC errors in LLD patients (i.e., $\rho_{\epsilon,1} =$ 0.1, 0.4, and 0.9, respectively), aimed to investigate the influence of $\rho_{\epsilon,1}$ specifically for alternative FC links on FDR control.

To illustrate the data structure in matrix notation, we can rewrite the bivariate linear mixed-effects model in Equation (2) for the $j^{th}$ subject using matrix notation as,

$$\boldsymbol{y}_j = \boldsymbol{X}_j\boldsymbol{\beta} + \boldsymbol{Z}_j\boldsymbol{\gamma}_j + \boldsymbol{\epsilon}_j, \tag{3}$$

where

- $\boldsymbol{y}_j$ is a $2m \times 1$ vector with the first $m$ elements for FC measurements and the last $m$ elements for SC measurements,

$$\boldsymbol{y}_j = \begin{bmatrix} y^{(F)}_{1j} & y^{(F)}_{2j} & \cdots & y^{(F)}_{mj} & y^{(S)}_{z,1j} & y^{(S)}_{2j} & \cdots & y^{(S)}_{mj} \end{bmatrix}^T_{1\times 2m}, \tag{4}$$

- $\boldsymbol{X}_j$ is a $2m \times 4m$ design matrix for the fixed effects $\boldsymbol{\beta}_k$,

$$
\begin{aligned}
\boldsymbol{X} &= \begin{bmatrix} 1 - Grp_j & \cdots & 0 & Grp_j & \cdots & 0 \\ \vdots & \ddots & \vdots & \vdots & \ddots & \vdots \\ 0 & \cdots & 1 - Grp_j & 0 & \cdots & Grp_j \end{bmatrix}_{2m\times 4m} \\
&= [1 - Grp_j \quad Grp_j] \otimes \boldsymbol{I}_{2m},
\end{aligned} \tag{5}
$$

  where $\boldsymbol{I}_{2m}$ is a $2m$-dimensional identity matrix.

- $\boldsymbol{\beta}$ is a $4m \times 1$ fixed effect vector,

$$\boldsymbol{\beta} = \begin{bmatrix} \boldsymbol{\beta}_0^{(F)T} & \boldsymbol{\beta}_0^{(S)T} & \boldsymbol{\beta}_1^{(F)T} & \boldsymbol{\beta}_1^{(S)T} \end{bmatrix}^T_{1\times 4m} \tag{6}$$

$$= \begin{bmatrix} \beta^{(F)}_{01} \cdots \beta^{(F)}_{0m} & \beta^{(S)}_{01} \cdots \beta^{(S)}_{0m} & \beta^{(F)}_{11} \cdots \beta^{(F)}_{1m} & \beta^{(S)}_{11} \cdots \beta^{(S)}_{1m} \end{bmatrix}^T_{1\times 4m} \tag{7}$$

- $\boldsymbol{Z}_j$ is a $2m \times 2$ design matrix for the random subject effects $\boldsymbol{\gamma}_j$,

$$\boldsymbol{Z}_j = \begin{bmatrix} \mathbf{1}_m & \mathbf{0}_m \\ \mathbf{0}_m & \mathbf{1}_m \end{bmatrix}_{2m\times 2}, \tag{8}$$

where $\mathbf{1}_m$ is a $m \times 1$ vector of all ones and $\mathbf{0}_m$ is a $m \times 1$ vector of all zeros.

- $\boldsymbol{\gamma}_j$ is a $2 \times 1$ vector of the random subject effects for FC and SC measurements,

$$\boldsymbol{\gamma}_j = \begin{bmatrix} \gamma_j^{(F)} \\ \gamma_j^{(S)} \end{bmatrix}, \tag{9}$$

- $\boldsymbol{\epsilon}_j$ is a $2m \times 1$ vector with the first $m$ elements for the random errors of FC and the rest $m$ elements for the random errors of SC,

$$\boldsymbol{\epsilon}_j = \begin{bmatrix} \epsilon_{1j}^{(F)} & \epsilon_{2j}^{(F)} & \ldots & \epsilon_{mj}^{(F)} & \epsilon_{1j}^{(S)} & \epsilon_{2j}^{(S)} & \ldots & \epsilon_{mj}^{(S)} \end{bmatrix}_{1\times 2m}^{T}. \tag{10}$$

For each scenario of correlation coefficients under each sample size of $n = 15$, 25, 35, and 45 subjects per group, 10,000 datasets are simulated. The FC and SC data in each simulation are analyzed separately using a mixed-effects model to compute the FC and SC test statistics for all the $m$ connectivity links, based on which the Lfdr using Efron's Lfdr and BLfdr using Bayesian multimodal Lfdr for each of the $m$ connectivity links are calculated. The oracle procedure described above is then applied to control FDR and determine which connectivity links are different between the two groups.

For the Bayesian multimodal Lfdr method, we run one MCMC chain for 20,000 iterations with the first 10,000 iterations discarded as burn-in and a thinning interval of 10 is used to produce a total of 1,000 random posterior draws for each parameter.

## REFERENCES

Bhaumik, D. K., Song, Y., Dan, Z., and Ajilore, O. A. (2018). Combining structural and functional neuroimaging data for studying brain connectivity: a review. *Journal of Biostatistics and Biometrics* 1, 1–10

Song, Y. (2016). *Sample Size Determination for High-Dimensional Neuroimaging Studies Controlling False Discovery Rate*. Ph.D. thesis, University of Illinois at Chicago

Zhao, W. (2014). *Statistical Methodologies for Group Comparisons of Brain Connectivity using Multimodal Neuroimaging Data*. Ph.D. thesis, University of Illinois at Chicago
